# Multimarker profiling identifies protective and harmful immune processes in heart failure: findings from BIOSTAT-CHF

**DOI:** 10.1093/cvr/cvab235

**Published:** 2021-07-15

**Authors:** George Markousis-Mavrogenis, Jasper Tromp, Wouter Ouwerkerk, João Pedro Ferreira, Stefan D Anker, John G Cleland, Kenneth Dickstein, Gerasimos Filippatos, Chim C Lang, Marco Metra, Nilesh J Samani, The BIOSTAT-CHF Consortium, Rudolf A de Boer, Dirk J van Veldhuisen, Adriaan A Voors, Peter van der Meer

**Affiliations:** Department of Cardiology, University Medical Center Groningen, University of Groningen, Hanzeplein 1, 9713 GZ Groningen, TheNetherlands; Department of Cardiology, University Medical Center Groningen, University of Groningen, Hanzeplein 1, 9713 GZ Groningen, TheNetherlands; Saw Swee Hock School of Public Health, National University of Singapore, 12 Science Drive 2, #10-01, Singapore 117549, Singapore; Duke-NUS Medical School Singapore, 8 College Road, Singapore 169857Singapore; Saw Swee Hock School of Public Health, National University of Singapore, 12 Science Drive 2, #10-01, Singapore 117549, Singapore; Department of Dermatology, Amsterdam UMC, University of Amsterdam, Amsterdam Infection & Immunity Institute, De Boelelaan 1117, 1118, 1081 HV Amsterdam, The Netherlands; Université de Lorraine, Inserm, Centre d'Investigations Cliniques, - PlurithÕmatique 14-33, and Inserm U1116, CHRU, F-CRIN INI-CRCT (Cardiovascular and Renal Clinical Trialists), Nancy, France; Cardiovascular Research and Development Center, Department of Surgery and Physiology, Faculty of Medicine of the University of Porto, Porto, Portugal; Division of Cardiology and Metabolism – Heart Failure, Cachexia & Sarcopenia, Department of Cardiology (CVK), Berlin-Brandenburg Center for Regenerative Therapies (BCRT), at Charité University Medicine, Charitépl. 1, 10117 Berlin, Germany; Department of Cardiology and Pneumology, University Medicine Göttingen (UMG), Robert-Koch-Straße 40, 37075 Göttingen, Germany; DZHK (German Center for Cardiovascular Research), Potsdamer Str. 58 10785 Berlin, Germany; Robertson Centre for Biostatistics, Institute of Health and Wellbeing, University of Glasgow, Glasgow G12 8QQ, UK; National Heart & Lung Institute, Imperial College, Guy Scadding Building, Dovehouse St, London SW3 6LY, UK; University of Bergen, Stavanger University Hospital, Gerd-Ragna Bloch Thorsens gate 8, 4011 Stavanger, Norway; Heart Failure Unit, Department of Cardiology, National and Kapodistrian University of Athens, School of Medicine, Athens University Hospital Attikon, Rimini 1, Chaidari 124 62, Athens, Greece; Division of Molecular & Clinical Medicine, University of Dundee, Dundee DD1 9SY, UK; Department of Medical and Surgical Specialties, Radiological Sciences and Public Health, Institute of Cardiology, University of Brescia, Piazza del Mercato, 15, 25121 Brescia BS, Italy; Division of Molecular & Clinical Medicine, University of Dundee, Dundee DD1 9SY, UK; Department of Cardiology, University Medical Center Groningen, University of Groningen, Hanzeplein 1, 9713 GZ Groningen, TheNetherlands; Department of Cardiology, University Medical Center Groningen, University of Groningen, Hanzeplein 1, 9713 GZ Groningen, TheNetherlands; Department of Cardiology, University Medical Center Groningen, University of Groningen, Hanzeplein 1, 9713 GZ Groningen, TheNetherlands; Department of Cardiology, University Medical Center Groningen, University of Groningen, Hanzeplein 1, 9713 GZ Groningen, TheNetherlands

**Keywords:** Inflammation, Heart failure, Immunomodulation, Biomarkers, Interferon-gamma, ICOSLG, CD28, CD70, TNFRSF14

## Abstract

**Aims:**

The exploration of novel immunomodulatory interventions to improve outcome in heart failure (HF) is hampered by the complexity/redundancies of inflammatory pathways, which remain poorly understood. We thus aimed to investigate the associations between the activation of diverse immune processes and outcomes in patients with HF.

**Methods and results:**

We measured 355 biomarkers in 2022 patients with worsening HF and an independent validation cohort (*n* = 1691) (BIOSTAT-CHF index and validation cohorts), and classified them according to their functions into biological processes based on the gene ontology classification. Principal component analyses were used to extract weighted scores per process. We investigated the association of these processes with all-cause mortality at 2-year follow-up. The contribution of each biomarker to the weighted score(s) of the processes was used to identify potential therapeutic targets. Mean age was 69 (±12.0) years and 537 (27%) patients were women. We identified 64 unique overrepresented immune-related processes representing 188 of 355 biomarkers. Of these processes, 19 were associated with all-cause mortality (10 positively and 9 negatively). Increased activation of ‘*T-cell costimulation*’ and ‘*response to interferon-gamma/positive regulation of interferon-gamma production*’ showed the most consistent positive and negative associations with all-cause mortality, respectively, after external validation. Within *T-cell costimulation*, inducible costimulator ligand, CD28, CD70, and tumour necrosis factor superfamily member-14 were identified as potential therapeutic targets.

**Conclusions:**

We demonstrate the divergent protective and harmful effects of different immune processes in HF and suggest novel therapeutic targets. These findings constitute a rich knowledge base for informing future studies of inflammation in HF.

## 1. Introduction

The pivotal role of the immune system in the initiation and progression of heart failure (HF) is supported by extensive literature.^1,^^2^ These findings have resulted in several studies on the effects of immunomodulatory therapies in HF, mostly focusing on tumour necrosis factor-α (TNF-α). The neutral or even negative results of these studies have fuelled the assumption that although HF is associated with increased immune activation, there might not be a causal relationship. However, the immune system is a highly complex entity incorporating interweaving molecular signalling mechanisms and numerous redundancies.^3^ An alternative hypothesis might thus be that past studies did not target the right immune processes and/or mediators. Hundreds of immune-related mediators take part in orchestrating an immune response,^3^ with some being used in revolutionary new treatments in the fields of immuno-oncology and rheumatology. As such, immunomodulation might still be a viable treatment option for HF. To identify such new targets in HF, a more holistic approach towards the study of immune-related biomarkers is required, as a single biomarker cannot realistically represent all aspects of the immune system. Therefore, the aim of this study was to characterize immune activation in a diverse cohort of patients with HF, in order to discern the differential effects of distinct immune-related processes on mortality and to identify promising targets for immunomodulation.

## 2. Methods

### 2.1 Patients

This was a *posthoc* analysis of the BIOSTAT-CHF study cohort, which has been described previously.^4^ Briefly, BIOSTAT-CHF was a multicentre observational study enrolling patients from 11 European countries; it was comprised of an index and validation cohort (*n* = 2516 and 1738, respectively). Participants in the index cohort were aged ≥18 years, had symptoms of new-onset or worsening HF, confirmed by a left ventricular ejection fraction (LVEF) ≤40% or brain-type natriuretic peptide (BNP) and/or N-terminal proBNP (NT-proBNP) plasma levels >400 or >2000 pg/mL, respectively. Participants had not been previously treated with angiotensin-converting enzyme inhibitors/angiotensin receptor blockers (ACEi/ARB) and/or β-adrenoreceptor blockers (BB) or were receiving ≤50% of guideline-recommended target doses, and anticipated their initiation or uptitration. All patients were treated with loop diuretics. The BIOSTAT-CHF validation cohort was designed as a multicentre, prospective, observational study including patients from six centres in Scotland, UK. Participants in the validation cohort were aged ≥18, were diagnosed with HF, had a previous admission for HF requiring diuretic treatment, were treated with furosemide ≥20 mg/day or equivalent, were not previously treated with or were receiving ≤50% of target doses of ACEi/ARB and/or BB, according to the 2008 European Society of Cardiology guidelines, and anticipated initiation or uptitration of ACEi/ARBs and/or BB. Patients could be enrolled as inpatients or from outpatient clinics. The primary outcome in both cases was all-cause mortality censored at 2-year follow-up. The study protocol conformed to the principles outlined in the declaration of Helsinki and was approved by local and national medical ethics committees (EudraCT 2010‐020808‐29; R&D Ref Number 2008‐CA03; MREC Number 10/S1402/39). All participants provided written informed consent before study inclusion.

### 2.2 Laboratory indices

We measured 368 biomarkers in plasma from 2022 and 1691 patients of the BIOSTAT-CHF index/validation cohorts (CVD-II/-III, immune and oncology panels; Olink Proteomics). Plasma was collected using calcium-ethylenediaminetetraacetic acid-coated tubes. Each panel included 92 biomarkers (listed in Supplementary material online, *Tables S1–S4*), with the only overlap being IL-6, c-kit ligand, and amphiregulin. For overlapping biomarkers, the mean of all measurements was used, leaving 364 distinct biomarkers. We also excluded 8 biomarkers with >10% of measurements below the assay’s lowest limit of detection (Supplementary material online, *Table S2*), leaving 356 biomarkers suitable for analysis. Other measurements included plasma concentrations of NT-proBNP, C-reactive protein (CRP), procalcitonin (PCT), high-sensitivity cardiac troponin-T (hs-cTnT), iron, ferritin, and transferrin. Estimated glomerular filtration rate was calculated using the MDRD formula. NT-proBNP, hs-cTnT, ferritin, and transferrin were measured using sandwich immunoassays (Roche Inc.), iron was measured using a colorimetric assay (Roche Inc.), PCT was measured using sandwich immunoassays (Alere Inc.), and CRP was measured using competitive immunoassays on a Luminex platform (Alere Inc.).

### 2.3 Statistical analysis

Statistical analyses were performed using R v.3.6.0 and the ‘GProfiler’ pathway analyser.^5^ Normality of continuous variables was determined using Q–Q plots/histograms. Normally distributed variables are presented as mean (standard deviation), continuous skewed variables are presented as median [interquartile range (IQR)], and binary/categorical variables are presented as number (%).

**Figure 1:**
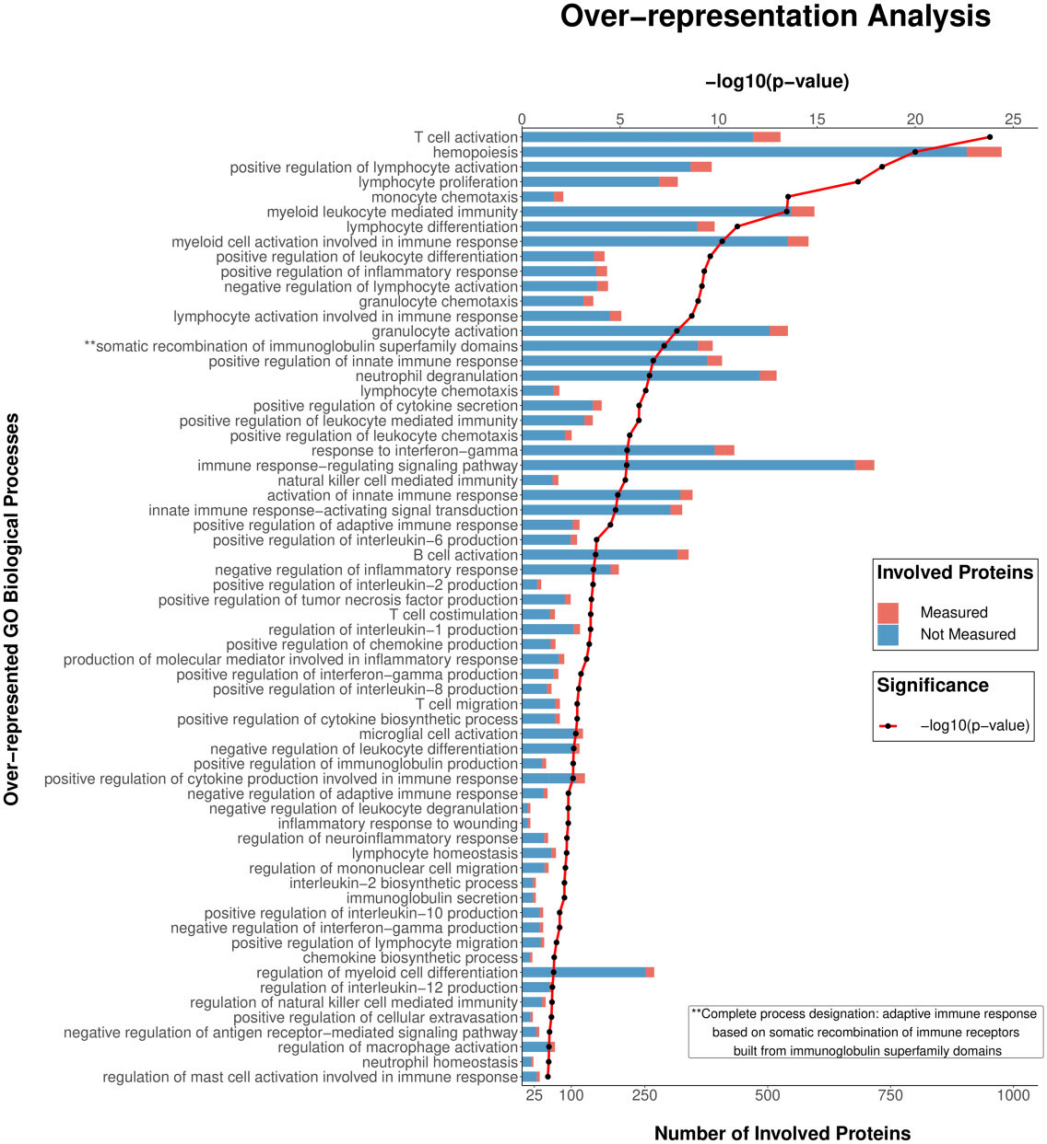
The 64 immune-related biological processes that were significantly overrepresented (*P*-value for overrepresentation analysis) based on 355 analysable biomarkers measured in 2022 and 1691 patients with heart failure from the BIOSTAT-CHF index and validation cohorts, respectively. Each bar denotes the total number of proteins involved in each process, with red denoting the fraction of proteins that were measured as part of the original plasma biomarker determinations. GO, gene ontology.

**Figure 2:**
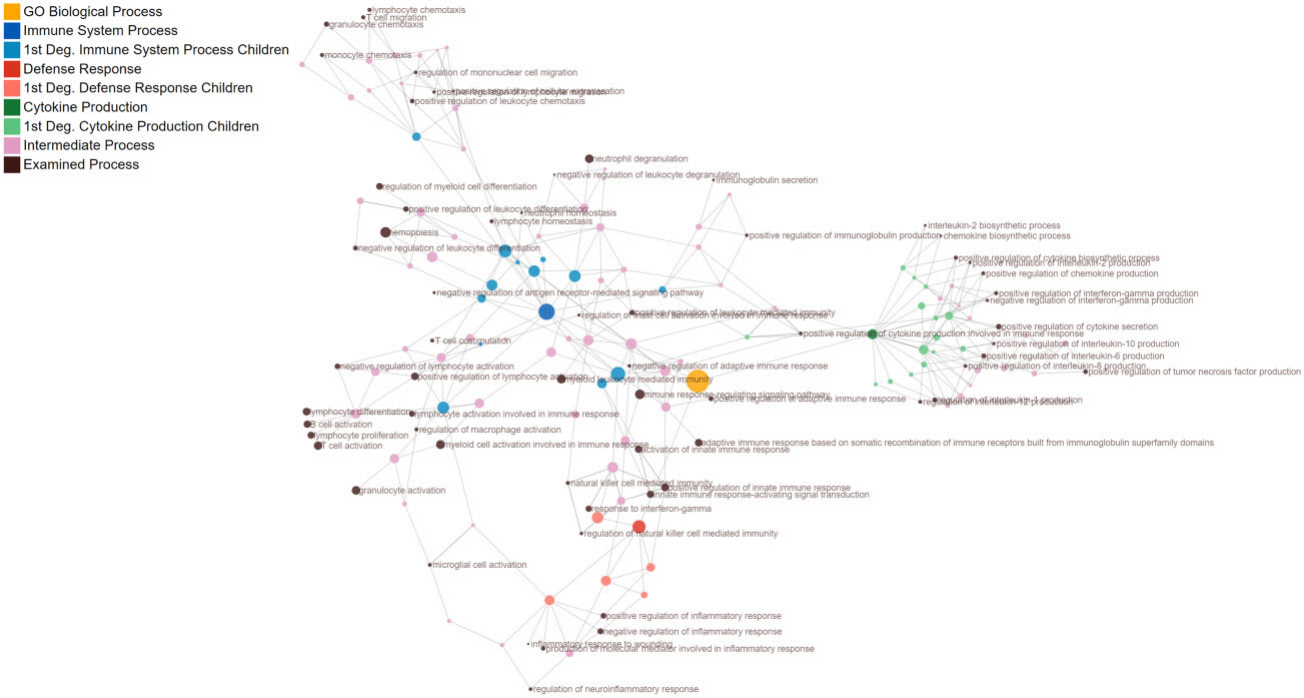
A directed acyclic graph showing the 64 examined immune-related processes and their parent processes (immune system process, defence response, cytokine production), based on the functions of 188 distinct biomarkers measured in 2022 and 1691 patients with heart failure from the BIOSTAT-CHF index and validation cohorts, respectively. Examined processes are denoted in brown and their parent terms are denoted either in blue, red, or green, respectively. First-degree children terms for parent process are shown in a lighter tone of the corresponding colour. Processes that are in-between first-degree children and the examined processes are denoted in pink. A fully interactive version of this graph including dynamic search capabilities for specific terms and on-click links to full process descriptions in the official GO website is provided in Supplementary material online, *Graphic S1*. GO, gene ontology.

Initially, the 356 analysable biomarkers were imported into GProfiler and an overrepresentation analysis was performed. To determine the functions of each biomarker, results were categorized based on the gene ontology (GO) classification of biological processes (annotation January 1 2020).^6,^^7^ Correction for multiple comparisons was performed using the built-in g:SCS algorithm (false discovery rate 5%); only processes with at least five of their constituents available were considered significant. Lastly, the biomarker corneodesmosin could not be analysed (355 biomarkers successfully analysed). In order to isolate only immune-related GO biological processes, we selected the most distant second- or third-degree children terms of the processes *cytokine production* (GO:0001816), *defenceresponse* (GO:0006952), and *immune system process* (GO:0002376) (*Figure 1 and 2* and Supplementary material online, *Graphic S1*, see also Supplementary material online, *Methods*).

To study immune-related biological processes, we utilized principal component analysis (PCA) to reduce the dimensionality of the biomarker constituents of each process. A weighted score (first principal component) was generated to which each biomarker contributed to a greater or lesser extent, based on how much population variance they explain. The weighted score for each process was used in multivariable Cox regression models to study their association with outcomes. The same procedure was followed in the validation cohort. The analysis of the index cohort was additionally corrected for antibiotic use. Proportionality of hazards was confirmed using standardized Schoenfeld residuals. Statistical significance was considered for *P*-value ≤0.05.

Selection of potential treatment targets was based on a two-pronged approach. The first criterion was individual biomarker membership only in processes significantly associated with all-cause mortality either negatively or positively; the most promising targets were selected based on their contribution to the particular process(es). The second criterion was biomarkers with large positive or negative net effects on mortality (i.e. biomarkers with contributions heavily favouring processes positively or negatively associated with all-cause mortality). In both cases, contributions refer to the extent each biomarker contributed to the weighted score of each process based on PCA. Biomarkers identified based on the first method are referred to as narrow-spectrum/high-specificity targets, while those identified based on the second method are referred to as broad-spectrum/low-specificity targets.

## 3. Results

Baseline characteristics for the index cohort are presented in *Table 1*. Mean age was 69 ± 12 years and 537 (27%) patients were women. Primary HF aetiology was most frequently ischaemic [895 (45%)], 202 (11%) patients had an LVEF >40%, and median NT-proBNP was 2679 pg/mL (IQR 1200–5639). At 2-year follow-up, 490 (24.3%) patients were rehospitalized for HF, and collectively 477 (23.6%) died of any cause; specifically, 316 (15.6%), 95 (4.7%), and 66 (3.3%) died due to CV, non-CV and unknown causes, respectively. Differences in baseline characteristics between the index and validation cohorts have been reported previously.^4^ In summary, compared with patients in the index cohort, those in the validation cohort were more often male, tended to be older, and had on average a higher LVEF and a larger proportion of LVEF >45%. In addition, they were more often recruited from the outpatient setting and had on average lower BNP and NT-proBNP values.

**Table 1:** Baseline characteristics of the total study cohort and stratified to tertiles of the weighted score for response to IFN-γ, the immune-related biological process with the strongest negative association with all-cause mortality

Variables	Total cohort	1st tertile of response to IFN-γ	2nd tertile of response to IFN-γ	3rd tertile of response to IFN-γ	*P*-value
Number of patients	2022	674	674	674	NA
Demographics					
Female sex	537 (26.6%)	184 (27.3%)	167 (24.8%)	186 (27.6%)	0.44
Age (years)	68.8 (12.0)	71.5 (11.4)	68.5 (12.0)	66.4 (12.2)	<0.001*
Years since 1st diagnosis of HF					
Clinical characteristics and comorbidities					
Primary HF aetiology					
Ischaemic	895 (45.1%)	318 (48.0%)	307 (46.6%)	270 (40.8%)	0.022*
Hypertensive	203 (10.2%)	72 (10.9%)	61 (9.3%)	70 (10.6%)	0.59
Cardiomyopathy	506 (25.5%)	134 (20.2%)	170 (25.8%)	202 (30.6%)	<0.001*
Valvular	161 (8.1%)	70 (10.6%)	44 (6.7%)	47 (7.1%)	0.018*
HF hospitalization in previous year	622 (30.8%)	242 (35.9%)	190 (28.2%)	190 (28.2%)	0.002*
Atrial fibrillation	918 (45.4%)	345 (51.2%)	322 (47.8%)	251 (37.2%)	<0.001*
Diabetes mellitus	645 (31.9%)	255 (37.8%)	203 (30.1%)	187 (27.7%)	<0.001*
Hypertension	1246 (61.6%)	448 (66.5%)	404 (59.9%)	394 (58.5%)	0.006*
Anaemia	708 (36.4%)	298 (46.1%)	216 (33.5%)	194 (29.8%)	<0.001*
COPD	346 (17.1%)	127 (18.8%)	109 (16.2%)	110 (16.3%)	0.34
Renal disease	575 (28.4%)	308 (45.7%)	167 (24.8%)	100 (14.8%)	<0.001*
Smoking					
None	736 (36.5%)	264 (39.2%)	226 (33.6%)	246 (36.5%)	
Past	988 (48.9%)	329 (48.9%)	335 (49.9%)	324 (48.1%)	0.075
Current	295 (14.6%)	80 (11.9%)	111 (16.5%)	104 (15.4%)	
NYHA functional class (prior to worsening HF)					
Class I	174 (10.0%)	42 (7.3%)	62 (10.7%)	70 (11.9%)	
Class II	931 (53.4%)	292 (50.5%)	304 (52.6%)	335 (57.1%)	<0.001*
Class III	571 (32.8%)	224 (38.8%)	181 (31.3%)	166 (28.3%)	
Class IV	67 (3.8%)	20 (3.5%)	31 (5.4%)	16 (2.7%)	
Physical examination					
BMI (kg/m^2^)	27.8 (5.5)	28.1 (5.6)	28.0 (5.5)	27.4 (5.3)	0.045*
Heart rate (beats/min)	80.1 (19.9)	80.2 (19.5)	79.8 (19.4)	80.3 (20.6)	0.88
Systolic blood pressure (mmHg)	124.8 (22.2)	123.5 (22.1)	125.4 (22.0)	125.5 (22.5)	0.20
Diastolic blood pressure (mmHg)	74.9 (13.3)	73.4 (13.3)	74.9 (13.4)	76.3 (13.2)	<0.001*
Rales/crepitation	1047 (53.3%)	390 (59.4%)	346 (52.7%)	311 (47.8%)	<0.001*
Echocardiographic indices					
LVEF (%)	30.0 (25.0–36.0)	30.0 (25.0–38.0)	30.0 (25.0–35.0)	30.0 (25.0–36.0)	0.16
LVEF > 40%	202 (11.2%)	83 (14.1%)	64 (10.6%)	55 (9.0%)	0.017*
Laboratory indices					
NT-proBNP (pg/mL)	2679.0 (1200.0–5639.0)	3898.5 (1777.0–8492.0)	2452.5 (1131.5–4974.0)	2080.0 (942.5–4284.0)	<0.001*
IL-6 (pg/mL)	5.1 (2.8–10.1)	6.6 (3.9–13.4)	5.1 (2.8–9.8)	4.0 (2.1–7.7)	<0.001*
CRP (mg/L)	13.4 (5.8–27.2)	17.5 (8.4–32.3)	13.1 (5.9–27.7)	10.4 (4.2–21.5)	<0.001*
High-sensitivity cardiac troponin-T (pg/mL)	31.3 (19.04–53.1)	41.5 (25.7–67.0)	29.5 (19.1–49.5)	25.1 (15.7–43.5)	<0.001*
eGFR (MDRD) (mL/min/1.73 m^2^)	63.7 (24.3)	52.6 (22.9)	65.1 (22.7)	73.5 (22.8)	<0.001*
Haemoglobin (g/dL)	13.2 (1.9)	12.8 (2.0)	13.3 (1.8)	13.4 (1.8)	<0.001*
Iron (μmol/L)	8.0 (5.0–12.0)	7.0 (5.0–11.0)	9.0 (5.0–13.0)	9.0 (5.0–13.0)	<0.001*
Ferritin (μg/L)	100.0 (49.0–190.0)	97.0 (52.0–190.0)	102.0 (52.0–196.0)	101.0 (43.0–183.0)	0.30
Transferrin (g/L)	2.0 (0.7)	2.0 (0.8)	2.1 (0.7)	2.0 (0.7)	0.068
Transferrin saturation (%)	16.8 (10.9–24.3)	15.5 (9.9–21.9)	17.4 (11.4–25.2)	18.2 (11.7–25.3)	<0.001*
Medications at baseline					
BB (baseline)	1680 (83.1%)	540 (80.1%)	567 (84.1%)	573 (85.0%)	0.038*
BB (target dose)	117 (5.8%)	39 (5.8%)	41 (6.1%)	37 (5.5%)	0.90
BB (% target dose)	0.3 (0.1–0.5)	0.3 (0.0–0.5)	0.3 (0.1–0.4)	0.3 (0.1–0.5)	0.71
ACEi (baseline)	1456 (72.0%)	444 (65.9%)	504 (74.8%)	508 (75.4%)	<0.001*
ACEi/ARB (target dose)	261 (12.9%)	73 (10.8%)	94 (13.9%)	94 (13.9%)	0.14
ACEi/ARB (% target dose)	0.3 (0.0–0.5)	0.3 (0.0–0.5)	0.3 (0.0–0.5)	0.3 (0.0–0.5)	<0.001*
MRA	1063 (52.6%)	326 (48.4%)	359 (53.3%)	378 (56.1%)	0.016*
Digoxin	375 (18.5%)	138 (20.5%)	120 (17.8%)	117 (17.4%)	0.28

ACEi, angiotensin-converting enzyme inhibitor; ARB, angiotensin receptor blocker; BB, β-adrenoreceptor blocker; BMI, body mass index; COPD, chronic obstructive pulmonary disease; CRP, C-reactive protein; eGFR (MDRD), estimated glomerular filtration rate calculated with the Modification of Diet in Renal Disease study group formula; HF, heart failure; IFN-γ, interferon-γ; IL-6, interleukin-6; LVEF, left ventricular ejection fraction; MRA, mineralocorticoid receptor antagonist; NA, not applicable; NT-proBNP, N-terminal pro-brain natriuretic peptide; NYHA, New York Heart Association.

* *P* ≤ 0.05 (also denoted in bold face).

### 3.1 Identification of immune system-related biological processes

Over-representation analysis of the 355 analysed biomarkers yielded 771 significantly over-represented biological processes. The selection of immune-related GO processes as described in Section 2 and the Supplementary material online, *Methods*, yielded after exclusion of 3 overlapping processes a total of 64 distinct immune-related biological processes. The 64 identified biological processes were represented by different combinations of 188 of the total 355 biomarkers in the overrepresentation analysis, and thus some biomarkers were constituents of more than one biological process (*Figure 2* and Supplementary material online, *Table S5*).

### 3.2 PCA and Cox regression

**Figure 3:**
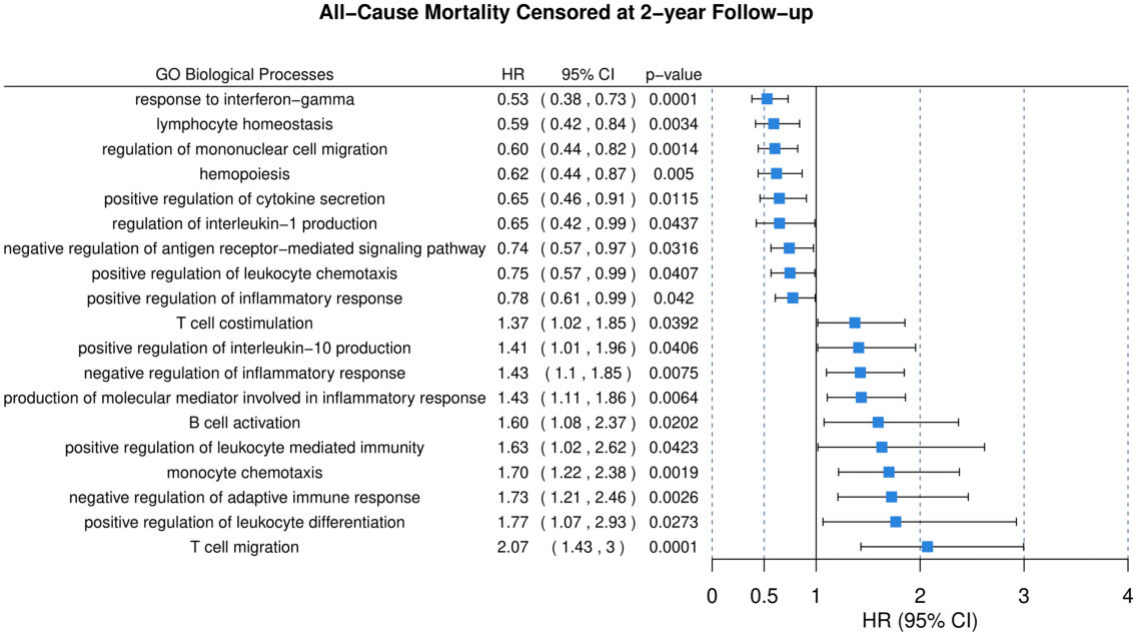
Multivariable Cox regression analysis of weighted scores of the 64 overrepresented immune-related biological processes. The analysis was carried out in 2022 patients of the BIOSTAT-CHF index cohort, as described in Section 2. Only significant processes (19/64) are presented. The complete overview of significant processes in the index and validation cohorts as well as their overlap are presented and classified by domain in *Table 2*. CI, confidence interval; HR, hazard ratio.

PCA was used to generate a weighted score for each of the 64 processes presented in *Figure 1*. A multivariable Cox regression analysis incorporating all processes, represented by their respective weighted scores, and corrected for known antibiotic use, identified 19 significant predictors of all-cause mortality at 2-year follow-up (9 negatively and 10 positively associated with all-cause mortality) (*Figure 3*). The omission of antibiotic use yielded almost identical results. Baseline characteristics were also stratified to tertiles of the weighted score for response to interferon-γ (IFN-γ), the immune-related biological process with the strongest negative association with all-cause mortality (*Table 1*). For brevity, biological processes with negative significant associations with all-cause mortality will henceforth be referred to as ‘protective’, while those with positive associations will henceforth be referred to as ‘harmful’. A number of additional sensitivity analyses were performed, where the model was corrected separately for age, sex, ischaemic aetiology, medication, and comorbidities. Most findings remained unaffected (Supplementary material online, *Figure S1*).

### 3.3 Independent validation

Independent validation of these results identified 6/19 processes also associated with all-cause mortality in the validation cohort (*Table 2*). When comparing the two cohorts, processes related to IFN-γ were highly protective in both, while T-cell costimulation had a shared harmful effect. B-cell-related processes were harmful in the index cohort but not in the validation cohort. Processes associated with all-cause mortality in the validation cohort are presented in Supplementary material online, *Figure S2*. Complete results for all 64 processes for the index and validation cohort are presented in Supplementary material online, *Tables S6 and S7*, respectively. Univariable Cox regression analysis for the 187 biomarkers involved in immune-related processes is presented for comparison in Supplementary material online, *Table S8*.

**Table 2:** Listing of biological processes that were significantly associated with all-cause mortality in the index cohort only, the validation cohort only, or both

Findings	Protective	Harmful	Process subfamily
Index cohort only (4)	Lymphocyte homeostasis^a^ Negative regulation of antigen receptor-mediated signalling pathway^a^	T-cell migration^a^ B-cell activation^a^	Adaptive immune response
Validation cohort only (3)	Positive regulation of immunoglobulin production^a^ Adaptive immune response based on somatic recombination of immune receptors built from immunoglobulin superfamily domains^a^	Regulation of natural killer cell-mediated immunity^a,^^b^	
Overlap (2)	NA	Negative regulation of adaptive immune response^a^ T-cell costimulation^a^	
Index cohort only (2)	Regulation of mononuclear cell migration^a^	Monocyte chemotaxis^a^	Innate immune response
Validation cohort only (2)	Regulation of myeloid cell differentiation^a^	Microglial cell activation^a^	
Overlap (0)	NA	NA	
Index cohort only (2)	Regulation of interleukin-1 production^c^	Positive regulation of interleukin-10 production^c^	Immune mediator production
Validation cohort only (4)	Positive regulation of interferon-gamma production^c^ Positive regulation of chemokine production^c^	Positive regulation of cytokine biosynthetic process^c^ Regulation of interleukin-12 production^c^	
Overlap (2)	Positive regulation of cytokine secretion^c^	Production of molecular mediator involved in inflammatory response^b^	
Index cohort only (5)	Response to interferon-gamma^a,^^b^ Haemopoiesis^a^ Positive regulation of leucocyte chemotaxis^a^	Negative regulation of inflammatory response^b^ Positive regulation of leucocyte-mediated immunity^a^	Other
Validation cohort only (0)	NA	NA	
Overlap (2)	Positive regulation of inflammatory response^b^	Positive regulation of leucocyte differentiation^a^	

Processes are presented in a simplified classification of whether they form part of the innate/adaptive immune response, those that are related to immune mediator production and others. Process membership based on the examined parent processes of ‘immune system process’, ‘defence response’, and ‘cytokine production’ is also provided.

NA, •••.

a Part of ‘immune system process’.

b Part of ‘defence response’.

c Part of ‘cytokine production’.

### 3.4 Characterization of biomarker functions

**Figure 4:**
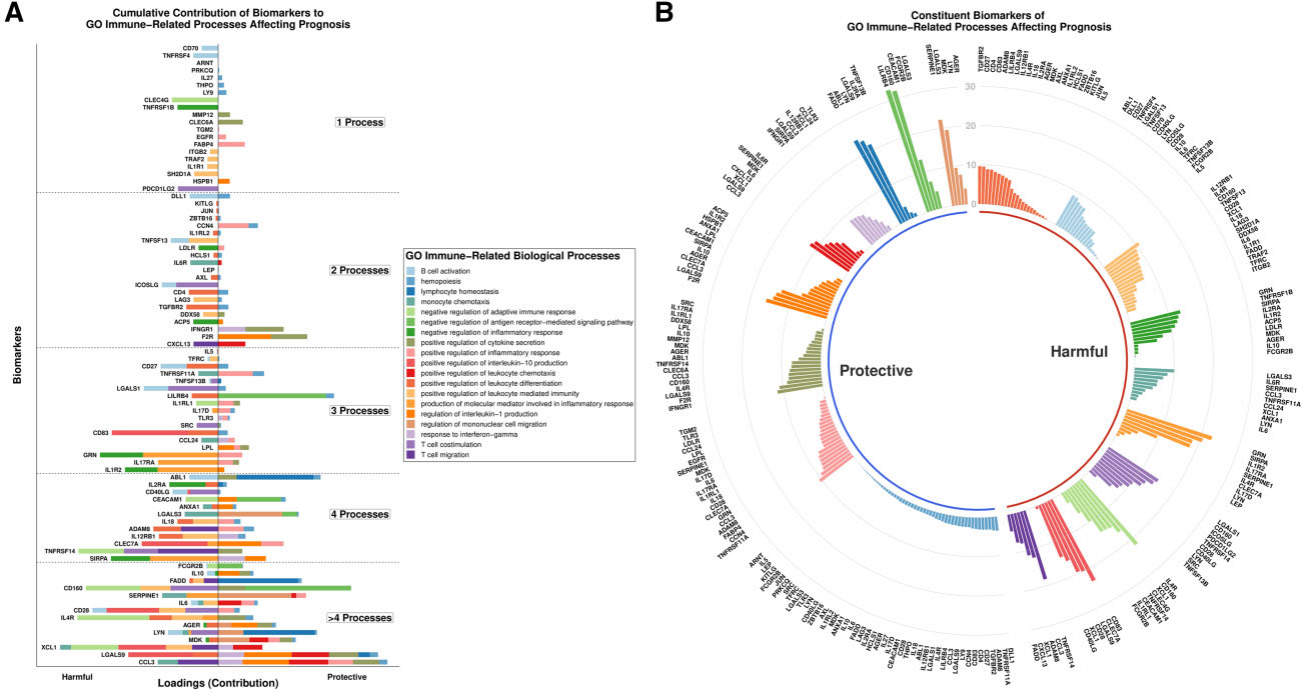
(*A*) Cumulative contribution of each biomarker to the weighted scores (principal components) of the 19 GO immune-related processes independently associated with all-cause mortality in the index cohort, sorted by the number of processes they are involved in. This analysis was carried out in 2022 patients of the BIOSTAT-CHF index cohort, as described in Section 2. Contributions to protective/harmful processes are on the right/left side of the graph, respectively. The dashed lines delineate biomarkers contributing to 1, 2, 3, 4, or >4 processes. (*B*) Circular bar plot displaying the contribution of individual constituent biomarkers to their respective processes, grouped by process and separated into protective and harmful categories. GO, gene ontology.

The contribution of each biomarker to the weighted score of the process/processes it constitutes was plotted only for processes significantly associated with all-cause mortality. For optimal visualization, only biomarkers that contribute to any significant processes in both the index and validation cohorts are shown. In total, 133 distinct biomarkers contribute to the 19 processes that were significantly associated with all-cause mortality in the index cohort (Supplementary material online, *Figures S3 and S4*). Of those, 84 biomarkers that also contributed to any significant processes in the validation cohort are shown in *Figure 4A and B*; the bars represent their relative contribution to each weighted score and have no meaningful unit of measurement. Most biomarkers contributed to both protective and harmful processes (59/84, 70%). The contributions of biomarkers to processes significantly associated with all-cause mortality in the validation cohort are presented in Supplementary material online, *Figures S5 and S6*.

### 3.5 Identification of potential therapeutic targets

#### 3.5.1 Narrow-spectrum/high-specificity targets

**Figure 5:**
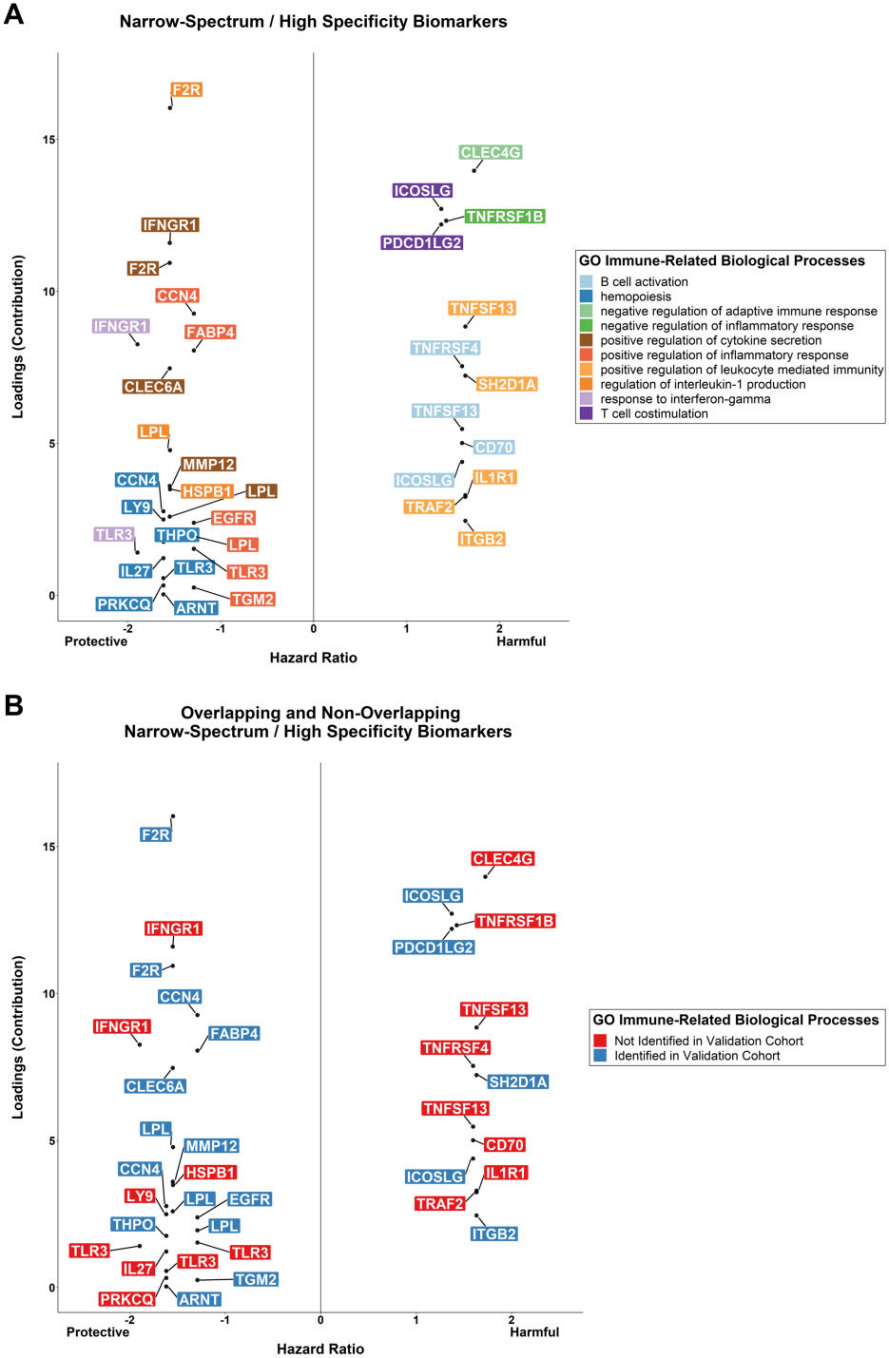
(*A***)** Biomarkers contributing only to the 9 protective or only to the 10 harmful immune-related processes presented in *Figure 3*, plotted by their contribution to and the hazard ratio of their respective process. These findings are based on the Cox regression analysis presented in *Figure 4* and carried out for 2022 patients with heart failure from the BIOSTAT-CHF index cohort. Hazard ratios for protective processes are presented as −1/HR. (*B*) Biomarkers that were and were not independently validated as contributors of only protective or harmful processes in 1691 patients of the BIOSTAT-CHF validation cohort. Biomarkers appearing >1 time, contribute to multiple processes.

First, to identify biomarkers that can serve as narrow-spectrum targets with high specificity for particular processes, we isolated those that contribute only to harmful or only to protective processes in both cohorts. Subsequently, their contributions were plotted against the hazard ratio of their corresponding process (*Figure 5A*). This allowed the stratification of biomarkers both by the prognostic significance of their underlying biological processes as well as by their relative contribution to those processes. Afterwards, the same graph was plotted but with the distinction between the finding being validated or not (*Figure 5B*); i.e. was the biomarker protective/harmful in both cohorts. Based on this, the most promising protective targets were thrombin receptor (F2R), cellular communication network factor 4, fatty acid-binding protein 4, lipoprotein lipase, and C-type lectin domain containing 6A, while the most promising harmful targets were programmed cell death 1-ligand 2 (PDCD1LG2), inducible costimulator ligand (ICOSLG), and SH2 domain-containing 1A.

#### 3.5.2 Broad-spectrum/low-specificity targets

**Figure 6:**
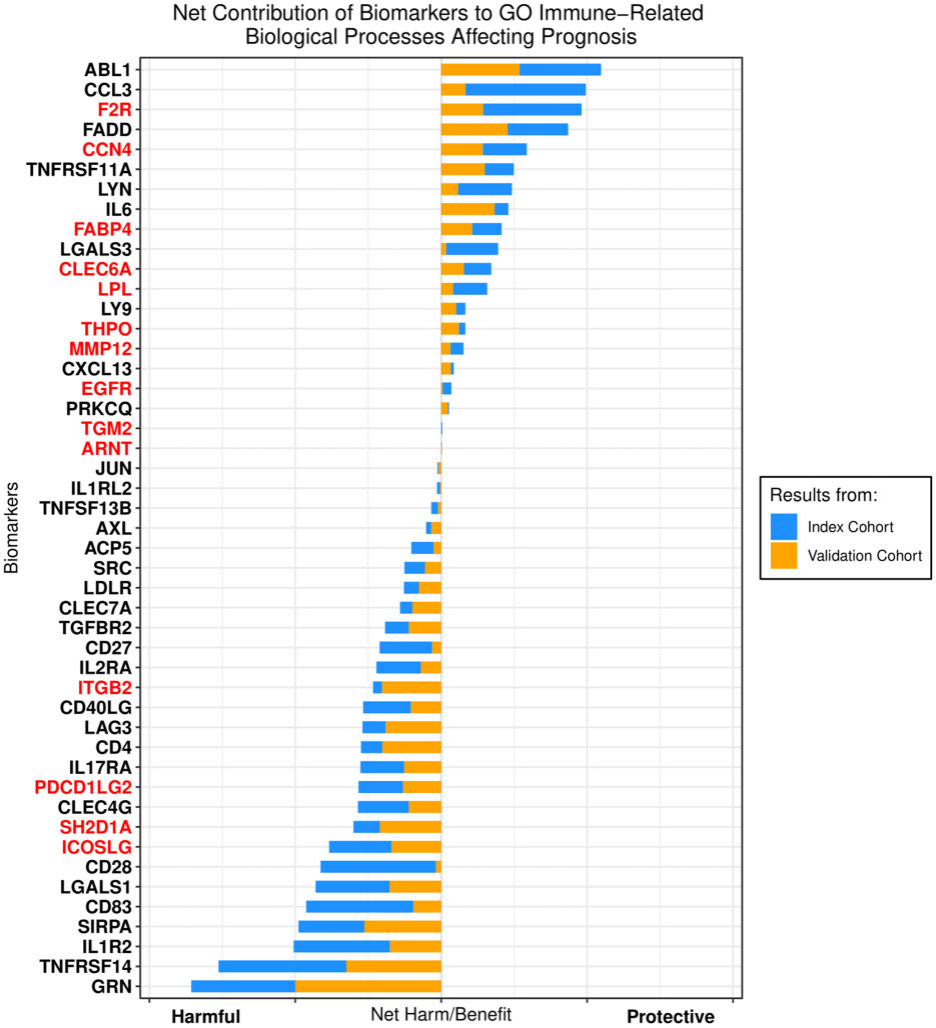
Net harm/benefit of biomarkers contributing to processes significantly associated with all-cause mortality both in 2022 patients in the index cohort and 1691 patients in the validation cohort. Biomarker names highlighted in red are only contributing to harmful or protective immune-related processes in both cohorts. GO, gene ontology.

Second, to isolate targets with the most positive and negative net/overarching effects, the net contribution of each of the 133 biomarkers was calculated by subtracting their collective contribution to harmful processes from their collective contribution to protective processes. Again, by only selecting biomarkers that behaved similarly in the index and validation cohort (net protective effect in both cohorts or net harmful effect in both cohorts), a stacked bar plot with the net contribution in each of the two cohorts was plotted (*Figure 6*). According to those results, the top three biomarkers with the greatest net harm were granulin precursor, TNF receptor superfamily member 14 (TNFRSF14), and IL-1 receptor 2, while those with the greatest benefit were ABL1, C-C motif chemokine ligand 3, and F2R.

## 4. Discussion

We present an extensive profiling of immune system activity in two independent, large and diverse cohorts of patients with HF. We demonstrate that biological processes related to production/response to IFN-γ are associated with a lower mortality, while processes related to T-cell activity are associated with a higher mortality. Individual biomarker analyses led to the identification of potential novel therapeutic targets which are described below.

The study of single biomarkers is often limited by confounding, some of which is accounted for in multivariable models. Nevertheless, the entirety of the immune system cannot realistically be modelled by studying a single representative biomarker.^3^ The novelty of our approach is that we used functional groupings of biomarkers instead of individual biomarkers, which allowed a more holistic profiling of immune-related processes. A particular biomarker may contribute both to protective and/or harmful processes, which is clearly illustrated by our data. Additionally, by including all over-represented biological processes in our multivariable prognostic model, we adjust individual processes for the relative state of activation of the remainder of the immune system. The advantages of this become clear when considering that the great majority of individual biomarkers are associated with worse outcomes (Supplementary material online, *Table S8*). Our data thus provide novel mechanistic insights as to the underlying immune-related processes that play a prominent role in HF, and constitute an extensive knowledge base for future studies.

IFN-γ is a cytokine with anti-viral, anti-neoplastic, and immunomodulatory properties,^8^ which can be both pro- and anti-inflammatory. Pro-inflammatory effects are more acute and include T-cell polarization to the Th1 subtype, inhibition of regulatory T cells (Tregs), and monocyte polarization to classical macrophages. In contrast, anti-inflammatory effects are more delayed and usually manifest in long-standing inflammatory states. These include inhibition of T-cell activity by promoting Treg proliferation and functions,^8–10^ and stimulation of the proliferation of myeloid-derived suppressor cells, which specifically inhibit T-cell activity.^11^ This is supported by the finding that increased T-cell activity is associated with higher all-cause mortality in both cohorts. Interestingly, *negative regulation of adaptive immune response* was associated with increased all-cause mortality in both cohorts. The immune system includes a multitude of regulatory negative feedback loops,^12^ which may become activated in case greater suppression is required. This might be a potential explanation for this finding. Additionally, since these data are derived from a multivariable Cox regression model, a process that might biologically be expected to be protective could appear harmful when the model is corrected for the relative activation state of the rest of the immune system. This is also supported by the fact that *positive regulation of cytokine secretion* and *positive regulation of inflammatory response* are protective in both cohorts. Lastly, the remaining two significant predictors of outcome for both groups, namely *positive regulation ofleucocytedifferentiation* and *production of molecular mediator involved in inflammatory response*, were both found to be harmful, which conforms with our expectations and results by others.^1,^^13,^^14^

A number of additional points merit further discussion in this context. The methodology that was followed relies on independent external validation of identified findings in the BIOSTAT-CHF index cohort. Thus, differences between the index and validation cohort^4^ could be seen as having major influence, seeing as concordance of findings between the two populations was a criterion for the selection of potential therapeutic targets. For instance, two related but different processes associated with IFN-γ (‘*response tointerferon-gamma/positive regulation ofinterferon-gamma production*’) were identified as significant predictors of the primary outcome in the index and validation cohorts and such differences could be attributed to the varying degree of HF severity and differing clinical characteristics between the index and validation cohorts. Of particular interest, patients in the index cohort were significantly younger than those in the validation cohort and were more often male. Differences in immune responses between sexes are apparent both throughout life as well as between puberty and menopause, thus suggesting that both genetic and hormonal influences are at work.^15^ In addition, processes such as immunosenescence and inflamm-ageing have received increasing scientific attention in recent years as major drivers of disease in the elderly and should thus not be underestimated as potential variables causing differences in identified processes between the index and validation cohort.^16^ Furthermore, the index and validation cohorts differed significantly in the proportion of patients with a preserved LVEF, and the validation cohort was comprised in general of patients with on average higher LVEF values. The pathophysiology and aetiology of HF with preserved and reduced LVEF is known to differ considerably between the two subtypes, and currently very little is known regarding differences in immune activation between the two.^17^ As such, this could be the focus of additional research focus in the future. Lastly, patients in the index cohort had on average significantly higher values of NT-proBNP compared with those in the validation cohort, which could reflect a greater clinical severity of HF in the former compared with the latter. This could also account for some of the identified differences. In general, the strength of the approach of independent validation is that it strengthens the generalizability and external validity of identified findings to other populations. Nevertheless, it could also be argued that certain processes were excluded due to the differences between populations. The remainder of the discussion will focus on describing potential novel therapeutic targets in patients with HF.

### 4.1 Therapeutic targets: IFN-γ

Historically, evidence has been equivocal regarding the cardiac effects of IFN-γ.^18^ More recently, two independent studies reported that IFN-γ^−^^/^^−^ mice subjected to pressure overload, developed more severe cardiac hypertrophy and had worse cardiac function.^19,^^20^ One of these studies also showed increased cardiac fibrosis in IFN-γ^−/−^ mice,^19^ while another demonstrated that IFN-γ promotes cell cycle arrest and induces an anti-fibrotic phenotype in human cardiac fibroblasts.^21^ Additionally, IFN-γ^−/−^ mice with experimental autoimmune myocarditis developed more severe disease^22^ and were more prone to transition to HF.^23^ IFN-γ also inhibits the production of IL-1 family cytokines. IL-1β and IL-18 are produced as inactive pro-IL-1β/pro-IL-18 and require proteolytic cleavage by the NLRP3 inflammasome to become active.^24^ IFN-γ inhibits NLRP3 inflammasome assembly by stimulating nitric oxide production,^24^ which is of particular relevance since the benefits of IL-1β blockade in myocardial infarction^25^ and potential benefits in HF^26^ have recently been demonstrated. Interestingly, stimulation of nitric oxide signalling with vericiguat reduced the combined endpoint of CV death and/or HF admission in patients with HF with reduced ejection fraction.^27^ NLRP3 inflammasome inhibition is also one of the postulated mechanisms by which sodium-glucose cotransporter-2 inhibitors exert beneficial CV effects.^28^ Enhanced IFN-γ activity might partially exert some of its protective effects in a similar manner. Our study thus supports the notion that enhancing IFN-γ production could constitute a potential therapy for HF. This is strengthened by the finding that patients with chronic HF have reduced circulating levels of IFN-γ compared with healthy controls, regardless of aetiology.^29^ Numerous studies have also reported a relationship between increased adrenergic activity and reduced IFN-γ production, which can be reversed by adrenergic blockade.^30,^^31^ This is particularly pertinent considering that BB are often prescribed for HF with known beneficial effects. It is also interesting to note that previous studies have reported that β-adrenoreceptor blockade can exert immunomodulatory effects in patients both with and without HF,^32,^^33^ although this cannot be directly corroborated by our findings.

### 4.2 Therapeutic targets: T-cell costimulation

To identify potential novel therapeutic targets, biomarkers were categorized into narrow- and broad-spectrum targets. Interestingly, a considerable proportion of either group consisted of biomarkers related to lymphocyte activation/costimulation. These included TNFRSF14, galectin-1 (LGALS1), ICOSLG, cluster of differentiation 40 ligand (CD40LG), PDCD1LG2, CD27, and CD28. Both T cells and B cells may recognize antigen via their T- and B-cell receptors. However, a second costimulatory signal (immune checkpoint) is required to prevent inappropriate activation. Costimulation provides survival signals for lymphocytes and promotes many of their functions. The aforementioned biomarkers usually exert their effects from their cell membrane, but they are also proteolytically cleaved by cell surface proteases or differentially spliced to produce soluble forms.^34,^^35^ These in turn are measurable in the blood, which can give an indication of their relative expression in the various immune cells. However, considering that only T-cell costimulation was a common predictive process in both the index and validation cohort, isolating targets belonging to that process might be the best approach. Of the aforementioned markers, ICOSLG and PDCD1LG2 were among the narrow-spectrum targets while TNFRSF14, LGALS1, CD27, CD28, and CD40LG were among the broad-spectrum targets.

ICOSLG primarily promotes the activation and function of effector T cells^36^ and plays an important role in cardiac immune responses, as ICOSLG produced by endothelial cells is increased during cardiac allograft rejection and stimulates cytotoxic T-cell responses.^37^ In addition, ICOSLG blockade halts progression of experimental autoimmune myocarditis in mice and reduces cardiac fibrosis.^38,^^39^ Notably, mice lacking functional T cells also do not transition from hypertrophy to HF after transverse aortic constriction.^14^ The monoclonal antibodies prezalumab and Rozibafusp alfa (AMG570) target ICOSLG and ICOSLG/B-cell activating factor, respectively.^40^ They have been studied in Phase II trials in Sjögren syndrome and systemic lupus erythematosus and might constitute potential treatments for HF. Potential pitfalls of this approach include the development of combined immunodeficiency after prolonged ICOSLG deficiency^41^ and the unintentional inhibition of Tregs, for which ICOSLG is also necessary,^36^ meaning that patient selection and treatment timing require careful consideration.

Apart from ICOS, the primary receptor for ICOSLG, CD28 also acts as a secondary receptor.^42^ CD28 is the main costimulatory molecule in T cells and is involved in four distinct harmful processes in our analysis. CD28 primarily binds to CD80/CD86 on antigen-presenting cells, which promotes T-cell activation. However, a related process called co-inhibition is mediated by cytotoxic T-lymphocyte-associated protein 4 (CTLA-4), which also binds to CD80/CD86 but has the opposite effect.^43^ Biologicals like abatacept and belatacept are recombinant CTLA-4 molecules attached to a human immunoglobulin tail and selectively bind to CD80/CD86. However, this might again negatively affect Tregs as CTLA-4 plays an important role in their function.^43^ More recently, there have been attempts to selectively target CD28, such that costimulation is prevented but co-inhibition remains unaffected. Two such biologicals, FR104 and lulizumab pegol, are in development and have shown safety and efficacy in a Phase I trial and a Phase II trial in systemic lupus erythematosus, respectively.^43^ Two Phase I/II trials with lulizumab pegol in allograft rejection are also currently underway. CD40LG induces B-cell activation and production of CD80/CD86;^44^ however, since B-cell activity was not uniformly protective or harmful, the benefits of CD40LG blockade can potentially be derived by selective CD28 blockade as mentioned previously.

Similarly to CD28, CD27 and its ligand CD70 control B- and T-cell function.^45^ Higher CD27/CD70 activity favours helper T-cell survival and induces apoptosis in Tregs.^46^ Interestingly, CD27^−^CD70^+^ Tregs paradoxically have pro-inflammatory effects, while CD27^+^CD70^−^ Tregs show strong inhibitory potential.^46,^^47^ Thus, modulation of CD27/CD70 signalling, particularly by selective inhibition of CD70 might be an attractive approach in HF. Lastly, PDCD1LG2 and LGALS1 are not optimal targets as they primarily inhibit T-cell activity.^48,^^49^ TNFRSF14 is involved in both pro- and anti-inflammatory activities via its non-redundant ligands TNF superfamily member-14 (TNFSF14) (pro-inflammatory), CD160 (mixed), and BTLA (anti-inflammatory).^50^ CD160 is also equally protective and harmful in our analysis. Thus, selective inhibition of TNFSF14 might be preferable to TNFRSF14 blockade.^51^

### 4.3 Considerations regarding potential therapeutic targets

Although the targets identified in this investigation present potential novel therapeutic opportunities for immunomodulation in patients with HF, care should be taken with potential clinical applications. In particular, immunomodulation is promising as a treatment because of the high degree of selectivity that can be achieved with specific inhibition or augmentation of molecular targets. At the same time, however, this can be a potential pitfall, as the multiple redundancies present within the immune system might circumvent the desired effect generated by the treatment. This consideration should be kept in mind when designing and investigating targeted therapeutics for specific molecular targets active within immune signalling. In addition, important considerations in this regard include the importance of patient selection, the time point of the initiation of treatment with targeted therapeutics, as well as the duration of treatment. In this respect, the findings of this study constitute a first step in the identification of potential targets, and further studies specifically in animals and patients with HF are necessary to elucidate the exact functions of each identified target, such that the aforementioned questions can adequately be addressed. The findings of this investigation constitute associations and not causative links; as such a specific biological process should be shown to be causally related to mortality to be able to draw definitive conclusions regarding therapeutic applications. Lastly, different aetiologies of HF might also have differential responses to targeted treatment and future investigations should take this into consideration. These considerations have been reviewed in detail recently.^52,^^53^

### 4.4 Limitations

Our study has a number of limitations. Although we present an extensive profiling of the immune system, this is based on a subset of processes represented by the available biomarkers. This affords a lesser degree of detail compared with a full-blood proteomics analysis. Additionally, physician-adjudicated infection at inclusion was not recorded. In the index cohort, this was partially resolved by correcting for current antibiotic use; however, this information was not available in the validation cohort. Furthermore, a potential limitation of this study is model overfitting due to the number of investigated biological processes. We were also unable to correct for HF duration. Future studies should also focus on longitudinal profiling of immune activation in order to account for temporal changes, as well as on investigating individual immune mechanisms in order to establish potential causative links between them and HF pathophysiology. Lastly, data on the prevalence of autoimmune rheumatic diseases and the use of immunomodulatory medication in the BIOSTAT-CHF cohort were not available.

## 5. Conclusion

In two large cohorts of patients with HF, profiling of immune system activity using a multimarker approach revealed immune-related biological processes associated with higher or lower all-cause mortality at 2-year follow-up. Biological processes related to T-cell costimulation and IFN-γ had the most important positive and negative associations with all-cause mortality, respectively. Potential therapeutic targets for future investigation include enhancing IFN-γ production and blockade of ICOSLG, CD28, CD70, and TNFSF14.

## Supplementary material

Supplementary material is available at *Cardiovascular Research* online.

## Authors’ Contributions

All authors met all four ICMJE criteria for authorship, gave final approval of the version to be published and agree to be accountable for all aspects of the work in ensuring that questions related to the accuracy or integrity of any part of the work are appropriately investigated and resolved. Specifically, regarding the following aspects, specific authors substantially contributed to: Conception/design: G.M.M., J.T., J.P.F., A.A.V., and P.v.d.M. Acquisition of data: S.D.A., J.G.C., K.D., G.F., C.C.L., M.M., N.J.S., R.A.d.B., D.J.v.V., A.A.V., and P.v.d.M. Analysis of data: G.M.M. and W.O. Interpretation of data: G.M.M., J.T., and W.O. Drafting the work: G.M.M., J.T., A.A.V., and P.v.d.M. Revising the work critically for important intellectual content: G.M.M., J.T., W.O., J.P.F., S.D.A., J.G.C., K.D., G.F., C.C.L, M.M., N.J.S., RA.d.B., D.J.v.V., A.A.V., and P.v.d.M.

**Conflict of interest**: A.A.V. received consultancy fees and/or research grants from Alere, Amgen, AstraZeneca, Bayer, Boehringer Ingelheim, Cytokinetics, Merck, Myokardia, NovoNordisk, Novartis, Cardio3Biosciences, Celladon, GSK, Merck, Novartis, Servier, Stealth Peptides, Singulex, Sphingotec, Trevena, Roche diagnostics, Vifor, and ZS Pharma. J.T. received consultancy fees from Roche diagnostics and personal fees from Olink proteomics. P.v.d.M. received consultancy fees and/or grants from Novartis, Corvidia, Singulex, Servier, Vifor Pharma, Astra Zeneca, Pfizer, and Ionis. J.G.C. reports personal fees from Abbott, grants and personal fees from Amgen, grants and personal fees from Bayer, personal fees and non-financial support from Medtronic, grants and personal fees from Novartis, grants and personal fees from Pharmacosmos, grants and personal fees from Vifor, grants and personal fees from BMS, and grants and personal fees from Servier, outside the submitted work. M.M. has potential conflicts of interest unrelated to this study: consulting honoraria from Bayer, Novartis, Servier as member of committees of clinical trials or advisory boards. S.D.A. reports grant support and personal fees from Vifor Int., grant support from Abbott Vascular, and personal fees from Astra, Bayer, Boehringer Ingelheim, Impulse Dynamics, Novartis, Respicardia, and Servier. J.T. has received speaker and/or personal fees from Roche diagnostics. R.A.d.B. received grants from AstraZeneca, Abbott, Boehringer Ingelheim, Cardior Pharmaceuticals GmbH, Ionis Pharmaceuticals, Inc., Novo Nordisk, and Roche and speaker fees from Abbott, AstraZeneca, Bayer, Novartis, and Roche. All other authors declared no conflict of interest.

## Funding

BIOSTAT-CHF was funded by the European Commission [FP7-242209-BIOSTAT-CHF; EudraCT 2010-020808-29]. The study was in part supported by a grant from the European Research Council (ERC CoG 818715, SECRETE-HF to R.A.d.B.).

## Data availability statement

Data are available on request.

## Supplementary Material

cvab235_Supplementary_Data

## Notes

Translational perspective Previous large randomized control trials employing agents targeting tumour necrosis factor-α in heart failure (HF) failed to show benefit. The current study serves as a knowledge base for future studies and drug development pipelines aimed at the identification of novel immunomodulatory agents or the repurposing of existing therapies for the treatment of HF. This is accomplished by a thorough multimarker mapping of immune activation in patients with HF and the identification of a multitude of novel targets that can be independently investigated.
