# Corrigendum to: Multimarker profiling identifies protective and harmful immune processes in heart failure: findings from BIOSTAT-CHF

**DOI:** 10.1093/cvr/cvac029

**Published:** 2022-04-09

In the originally published article, the provided supplemental figures and tables were erroneously missed in being uploaded to the Supplementary Data file. The figures and tables have now been added to the Supplementary Data file online.

