# Supplementary material for: Multimarker profiling identifies protective and harmful immune processes in heart failure: findings from BIOSTAT-CHF
Source: Cardiovasc Res. 2021 Jul 15;118(8):1964–77. doi: 10.1093/cvr/cvab235 (PMC9239579; doi:10.1093/cvr/cvab235)
Supplement: cvab235_Supplementary_Data [file cvab235_supplementary_data.zip › Supplementary Graphic 1.html]

Locate

Reset Network

- **Directed acyclic graph of over-represented GO immune-related biological processes**
- G. Markousis-Mavrogenis, et al. 2021; The BIOSTAT-CHF study
