# Supplementary material for: Multimarker profiling identifies protective and harmful immune processes in heart failure: findings from BIOSTAT-CHF
Source: Cardiovasc Res. 2021 Jul 15;118(8):1964–77. doi: 10.1093/cvr/cvab235 (PMC9239579; doi:10.1093/cvr/cvab235)
Supplement: cvab235_Supplementary_Data [file cvab235_supplementary_data.zip › BIOSTAT Vanguard-Suppl. Materials_CVR Submission.pdf]

Supplementary Materials

Multimarker Profiling Identifies Protective and Harmful Immune Processes in Heart Failure: Findings from BIOSTAT-CHF

G. Markousis-Mavrogenis et al., Cardiovascular Research 2021

Supplementary Methods

Technical Considerations Regarding Statistical Analyses

For this study we initially employed 364 distinct biomarkers. These were subsequently filtered based on whether or not they take part in biological processes related to the immune system, as well as the quality of the individual biomarker measurements, as described in the main manuscript. Nevertheless, even after filtering we were still left with 188 biomarkers that could potentially predict mortality, still complicating statistical analyses. The reason why this is the case is because an individual biomarker may take part in different biological processes with some even contributing to the same processes. Thus, the degree of potential multicollinearity in this dataset was deemed to be quite high. Multicollinearity is defined as "the existence of such a high degree of correlation between supposedly independent variables being used to estimate a dependent variable, that the contribution of each independent variable to variation in the dependent variable cannot be determined" [1]; or in more simple terms, that there is overlap in the predictive capacity of independent variables with regard to a dependent variable, in this case the biomarkers and patient prognosis respectively. In order to solve this issue, we employed a combined approach using classification of biomarkers based on their functions according to the Gene Ontology (GO) classification system, followed up by dimensionality reduction based on principal component analysis. The tools and principles behind them will now be described in detail in the following sections.

The Gene Ontology Classification

The Gene Ontology (GO) classification is a powerful tool uniting current knowledge on the function of different proteins in the human body. It is a curated database that is updated frequently to include new findings and better understanding of human biology [2,3]. The GO classification has the form of a directed acyclic graph (DAG), that is, a form of causal graph that associates events with other events [4]. The term directed refers to the property of symbolizing the associations between different parts of a causal network using arrows pointing from cause to effect. The term acyclic refers to the fact that no path of directed arrows can form a closed loop. The discerning reader might notice that the directed arrows in the DAG presented in **Figure 1A** of the main manuscript and the corresponding supplementary interactive graphic point outwards toward the examined processes and not inwards as would be expected based on this definition. This was done solely for the purposes of easier visualization, as it was deemed that the graph was more readable in this format. In essence, **Figure 1A** has the appropriate hierarchical format of a DAG with only difference being that the directed arrows point in the opposite direction, from effect (i.e. Biological Process) to cause (i.e. examined processes). This distinction aside, **Figure 1A** is a typical example of a DAG structure. A DAG with arrows pointing in the appropriate direction is presented in **Supplementary Methods Figure 1** (the figure is adapted from the GO official website accessible at http://geneontology.org/docs/ontology-documentation).

The GO classification offers three main options for classifying a selection of proteins, namely classification either by cellular component (e.g. nucleus, cell membrane, etc.), molecular function (e.g. catalysis, transport, etc.), or biological process. We opted for the latter classification as it allowed us to better discern which biomarkers were involved in processes related to the immune system. As described in the main manuscript, an over-representation analysis was performed based on the GO classification, to identify biological processes overrepresented by the available biomarker selection. Since GO has the form of a DAG, higher level processes collectively contain "more

information" than lower level processes, as is the case in e.g. **Supplementary Methods Figure 1** for *metabolic process*. After visual inspection of high-level terms in the GO classification, a selection was made for *immune system process*, *defense response* and *cytokine production* as the GO biological processes most related to the immune system. Each of these processes (termed parent processes) has a number of children terms (see also **Figure 1A** in the main manuscript). All of the children terms that belonged to the aforementioned parent processes and were significantly overrepresented by the available biomarkers were isolated for further refinement before continuing with analyses.

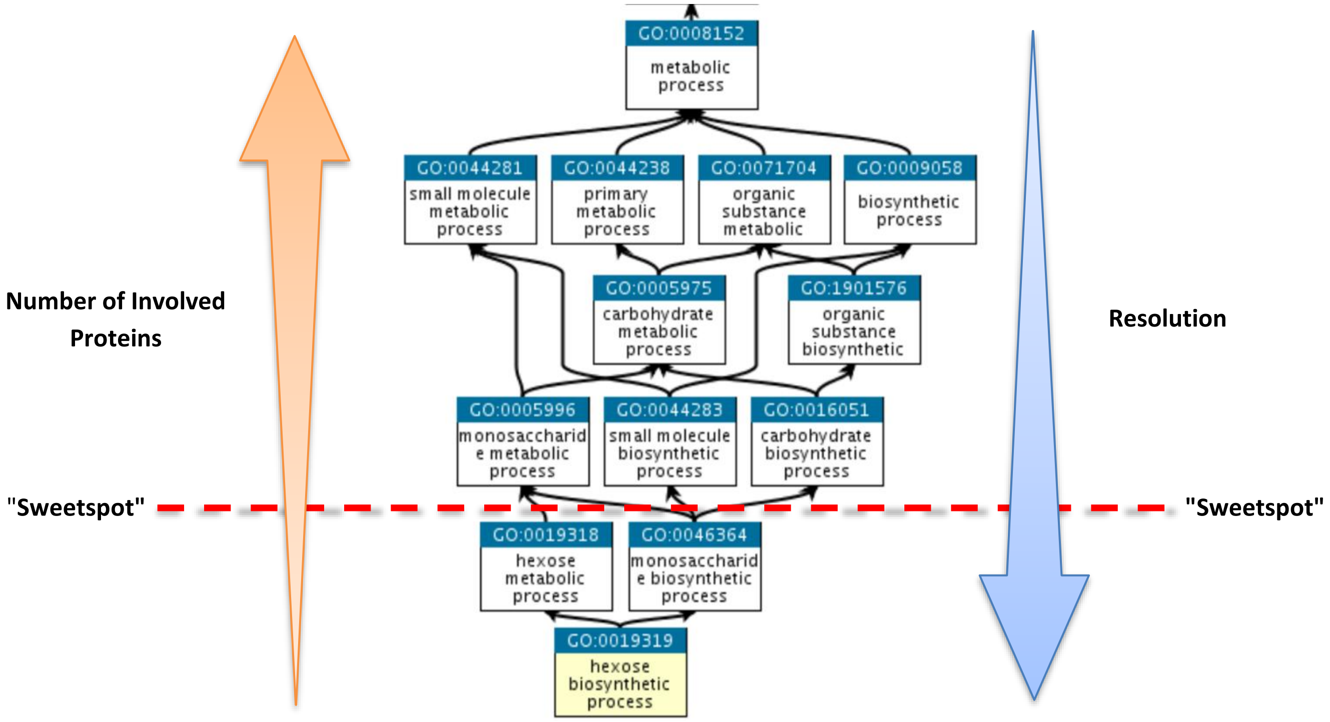

Supplementary Methods Figure 1. A typical directed acyclic graph in the Gene Ontology classification. Higher-up (parent) processes like *metabolic process* contain all the protein members of processes that are connected to them with directed arrows. The colored arrows on the sides represent the inverse proportion between the number of involved proteins and the provided resolution when moving up and down through the classification. "Sweetspot" refers to the ideal tradeoff between high resolution and not too low number of involved proteins.

Considerations Regarding the Application of the Gene Ontology Classification

However, there are some considerations that need to be taken into account when GO is used for data analysis:

1) Firstly, one has to be aware of the fact that there is a certain degree of redundancy in every protein, meaning that within the GO classification of biological processes it can be part of a multitude of related or unrelated processes. This is tackled using dimensionality reduction within each examined biological process and is described in the following section. In order to be able to apply any dimensionality reduction techniques however, we first need to identify which processes will be included in the analysis.

2) Thus, secondly, the issue of resolution and its associated diminishing returns need to be taken into account. What is meant by this is that, in the example of **Supplementary Methods Figure 1**, the top process

(*metabolic process*) contains all of the processes below it as well as their member proteins. In an overrepresentation analysis, if the bottom process (*hexose biosynthetic process*) is significantly overrepresented then the same is the case for all its parent terms, thus leading to a series of overrepresented processes with sequentially fewer protein members from top to bottom. It is thus pertinent to consider what degree of detail or resolution we might be interested in, as it can be expected that the deeper into individual processes we go, the more diminishing returns we get. That is to say, an overrepresented process might for example have only 3 or 4 biomarker members which is marginally better than using the individual markers themselves (dimensionality reduction is highly redundant when dealing with very small numbers of variables). There is thus a tradeoff where high level processes contain too many proteins to offer any meaningful resolution while low level processes offer too much resolution, to the point of being redundant. It is then up to the individual researcher to select the appropriate level (sweetspot) of detail that is required in a particular study. In our case, we arbitrarily chose to include the most distant 1st, 2nd or 3rd degree relatives of the three aforementioned selected parent processes. By "degree", it is implied that a 1st degree relative is directly connected to the parent process with an arrow, a 2nd degree relative is connected to the parent process via an intermediary and a 3rd degree process is connected to the parent process via two intermediaries.

3) Thirdly, there is the matter of the innate redundancy of regulatory processes. In the GO classification of biological processes, regulation of a process may belong either to a process entitled "*positive regulation of […]*" or "*negative regulation of […]*". Both of these are connected to the process they regulate but also to a collective regulation term ("*regulation of […]*"). As can be seen is **<u>Supplementary Methods Figure 2</u>**, the positive and negative regulation terms can be both 1st and 2nd degree relatives of the same process and are overlapping with the collective regulation term that contains all of their individual members. This needs to be taken into account when selecting individual processes. The following section deals with how considerations 2 and 3 were dealt with and is followed up by additional information on dimensionality reduction and principal component analysis (consideration 1).

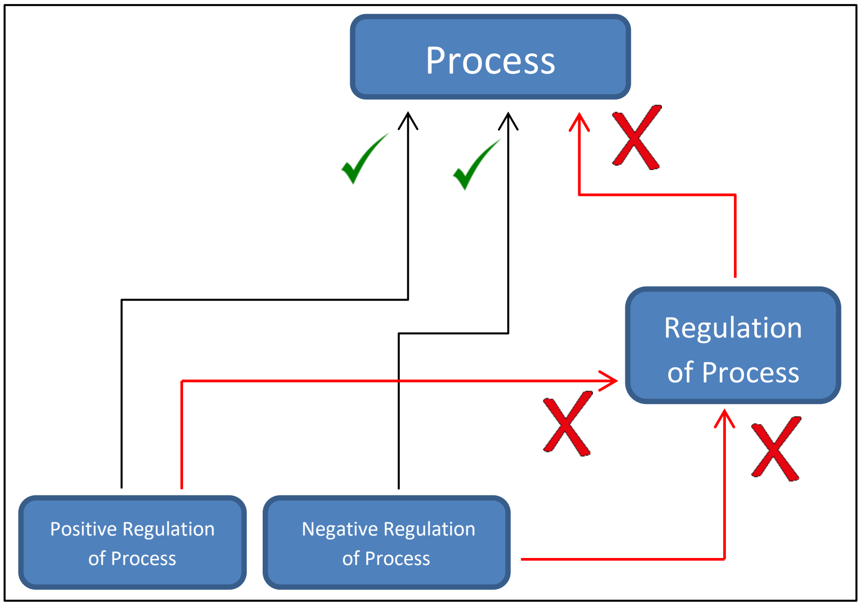

Supplementary Methods Figure 2. The relationship of positive and negative regulation terms with the process they regulate and the collective regulation term. relationships denoted with a green tick are those we are interested in, while those denoted with a red cross are redundant are were excluded.

Method of Process Selection

As discussed in the main manuscript, we only examined overrepresented processes with at least 5 of their members present in our biomarker selection (this with regard to the aforementioned issue of resolution). We subsequently isolated the most distant 1st, 2nd and 3rd degree relatives of the selected parent terms for further analyses. We will now illustrate how this and the subsequent final selection of processes was done exactly. As mentioned in the previous section, in an overrepresentation analysis, when a process is significantly overrepresented, the same holds true for all its parent processes in the GO classification. However, there was also the issue of the regulation terms as described in the previous section and shown in **Supplementary Methods Figure 2**. With that in mind, we analyzed the results of the overrepresentation analysis we performed in a specific manner. Namely, starting with the 1st degree relatives of the three main parent processes we selected, we investigated in a stepwise manner which of those have significantly overrepresented children terms, up to and including children terms of 4th degree (**Supplementary Methods Figures 3, 4, 5**). To identify collective regulation terms that should be excluded, we took advantage of the fact that positive and negative regulation terms are both 1st and 2nd degree relatives of the processes they regulate (**Supplementary Methods Figure 2**). Specifically, we selected only processes that had overrepresented children which were the same degree relatives as the processes themselves in order to identify redundant regulation terms. This is charcteristically seen in **Supplementary Methods Figure 5**, where there are two 3rd degree children terms, one with an overrepresented child term and one without, but no 4th degree children terms, clearly suggesting that the child is at the same level as the parent. Indeed, the two processes are *regulation of tumor necrosis factor production* and *positive regulation of tumor necrosis factor production*, of which the former was thus identified and excluded. Using this procedure, we identified 67 2nd or 3rd degree children terms of the three originally selected parent terms. After removal of 3 duplicate processes, a total of 64 unique and non-redundant processes were identified, which were subsequently used to apply dimensionality reduction techniques.

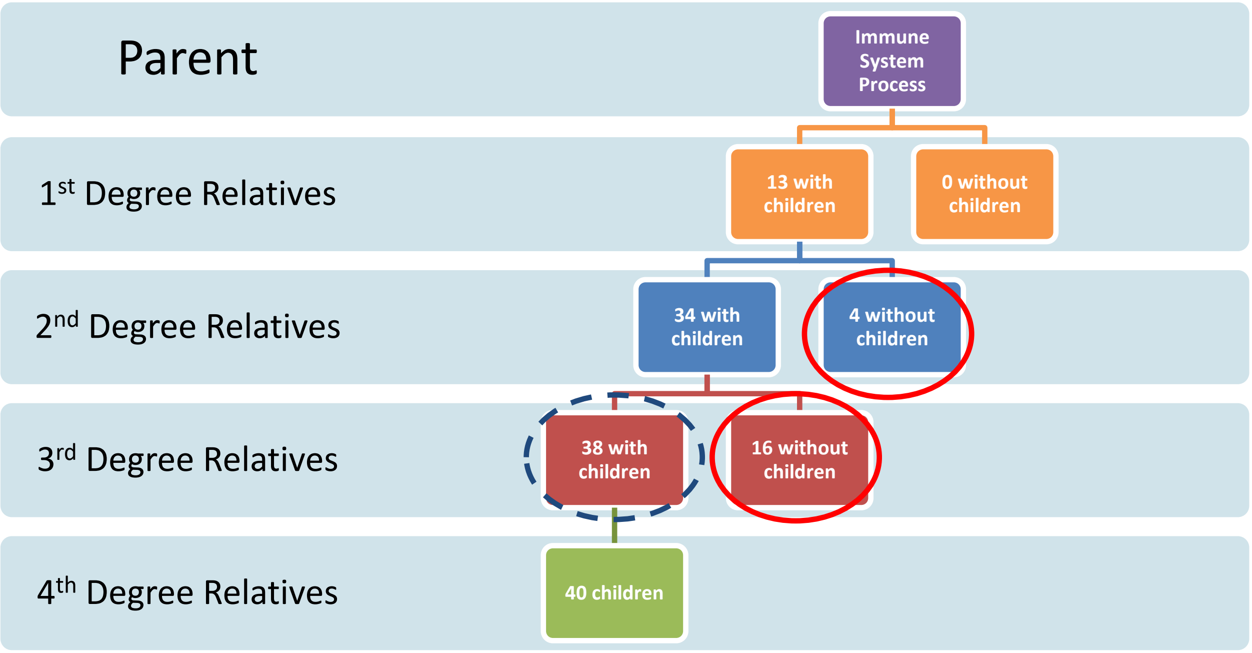

Supplementary Methods Figure 3. Analysis of GO biological processes with and without children terms that were relative of the parent process entitled "*immune system process*". Processes circled in red are included in the analysis as they are; processes circled in dark blue are first examined for redundant processes as described in the text. GO gene ontology

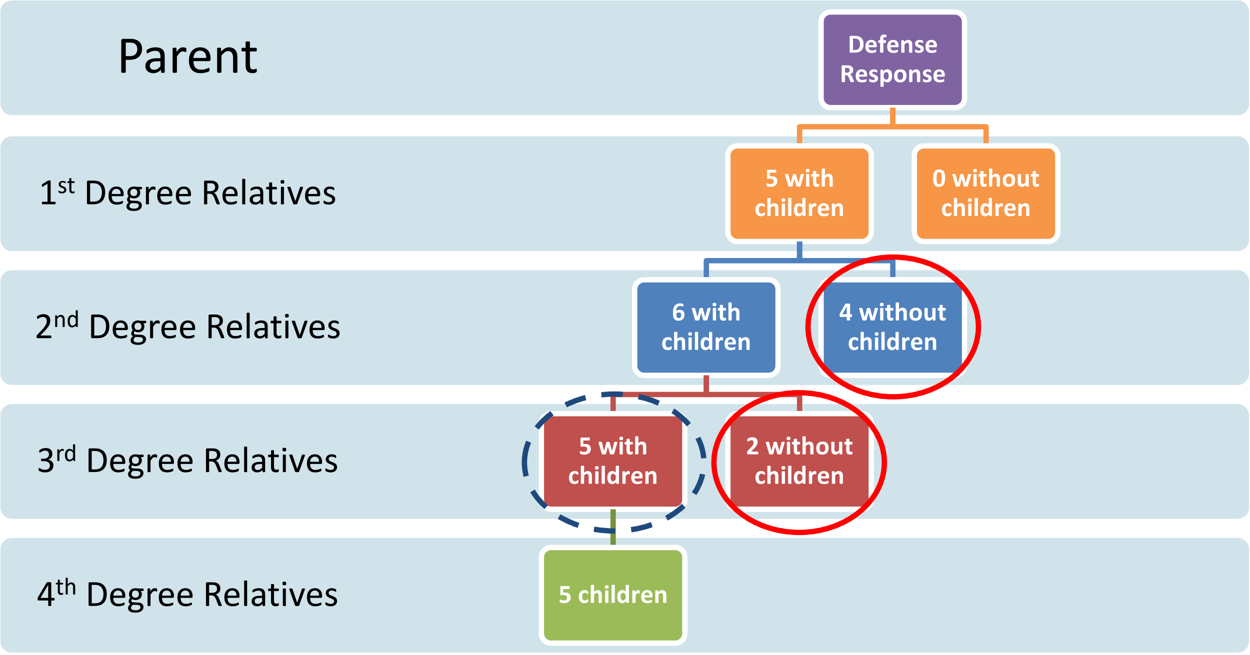

Supplementary Methods Figure 4. Analysis of GO biological processes with and without children terms that were relative of the parent process entitled "*defense response*". Processes circled in red are included in the analysis as they are; processes circled in dark blue are first examined for redundant processes as described in the text. GO gene ontology

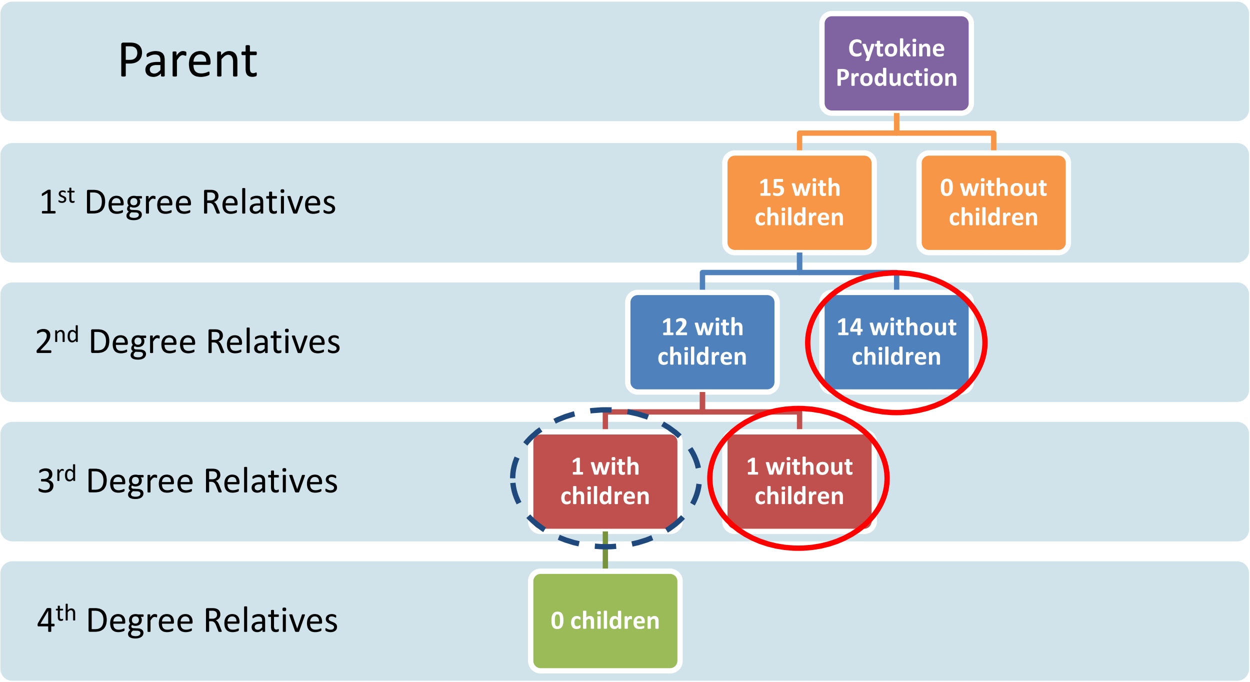

Supplementary Methods Figure 5. Analysis of GO biological processes with and without children terms that were relative of the parent process entitled "*cytokine production*". Processes circled in red are included in the analysis as they are; processes circled in dark blue are first examined for redundant processes as described in the text. GO gene ontology

A Primer on Dimensionality Reduction

Having finalized the selection of GO biological processes to be analyzed, a way had to be identified to be able to represent these biological processes in a regression model with a single variable instead of their individual constituent biomarkers, as some biomarkers may be part of multiple processes at the same time and there is also the issue of multicollinearity as presented in the beginning of the supplementary methods. In addition, a regression model with too many variables faces issues such as overfitting, that is, the model predicts the dependent variable too well in the examined dataset but is not easily generalizable to other datasets [5].

In essence, representing the same amount of information with fewer variables is what is referred to as "dimensionality reduction" in statistical terms [6,7]. Dimensionality reduction is an evolving field that is becoming more and more relevant in the era of big data. There are different ways to perform dimensionality reduction, among others including methods that select a subset of the most representative variables from a bigger whole (feature selection) and those that generate a new set of fewer variables based on a combination of the input data that contains the same information as the input data (formally called dimensionality reduction) [6,7]. An example of a feature selection method is penalized regression analysis and random forests [6,8]. Projection methods for dimensionality reduction include t-stochastic neighbor embedding (t-SNE) which is commonly used in single-cell RNA sequencing studies, but also the related method called principal component analysis which was used in our study. In this case, we chose for the latter and specifically principal component analysis, as it allows us to preserve all the available information, we have instead of eliminating less important variables from the analysis.

The Concepts Behind Principal Component Analysis

The principles behind how principal component analysis works require detailed description and have been the subject of a recent publication by Jolliffe et al. [9]. A less technical description of the steps of principal component analysis is also presented elsewhere, should the reader be interested [10]. Thus, these will not be the focus of this document. Principal component analysis was first described by Pearson and Hotelling in 1902 but has seen a resurgence in the era of big data and sufficient computer processing power. The notion behind principal component analysis is simple, namely to "reduce the dimensionality of a dataset, while preserving as much variability (i.e. statistical information) as possible" [9]. In other words, we want to convey the same amount of information but with fewer variables. Before concluding with this supplementary description of the methods, it is important define the statistical term "variance".

- **Variance** is defined as the measure of how a set of random numbers are spread out from their average value. The formula for determining variance depends on the distribution of the variable (e.g. normal Gaussian, binomial, exponential, etc.) but the principle remains the same.

Using principal component analysis, we can generate a set of new variables (principal components) that explain the same amount of variance in our dataset as a similar set of biomarkers but with fewer overall variables. By definition, the 1st principal component always explains the greatest amount of variance. This makes it ideal for use as a single variable to represent a particular biological process. Biomarkers were analyzed according to the results of the overrepresentation analysis in groups, based on the processes they contribute to (biomarkers contributing to multiple processes were included in all corresponding analyses). All the biomarkers taking part in a particular biological process contribute to a greater or lesser extent to the 1st principal component of the processes they take part in, thus allowing us to stratify the relative importance of each biomarker in patients with heart failure and to circumvent the issue of multicollinearity. A summary of the analysis pipeline is presented in **Supplementary Methods Figure 6**.

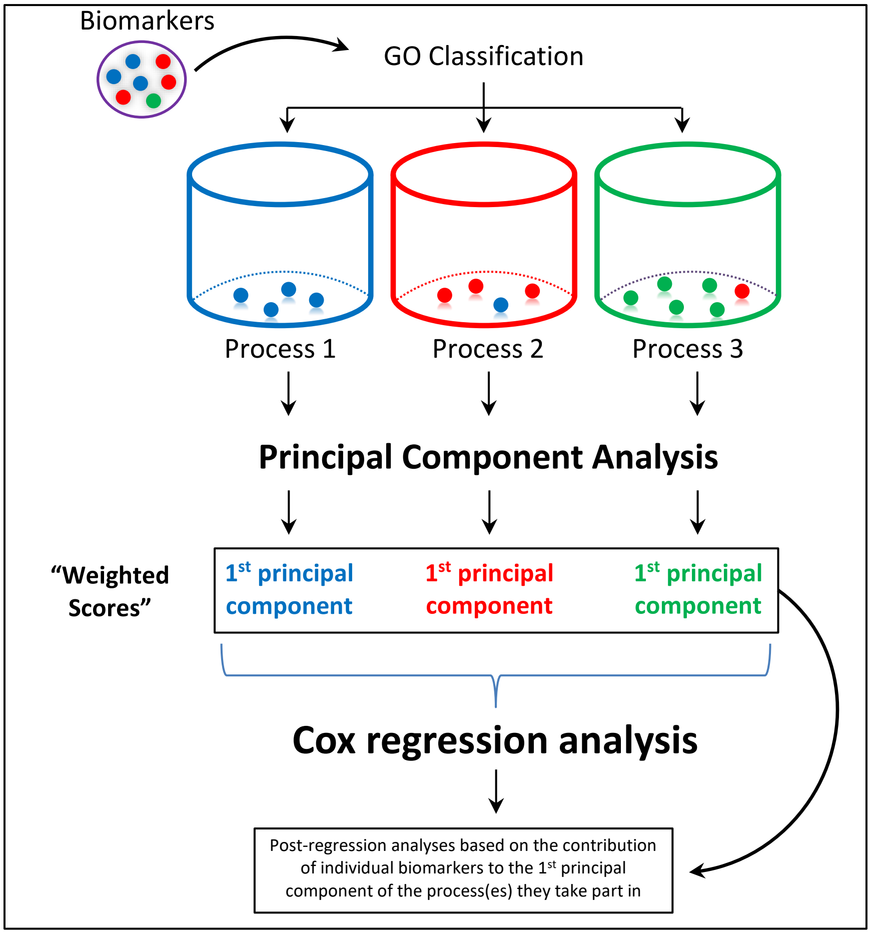

Supplementary Methods Figure 6. A summary of the analysis process as performed in the main manuscript.

References

1. Multicollinearity | Definition of Multicollinearity by Merriam-Webster. https://www.merriam-webster.com/dictionary/multicollinearity (15 June 2020)

2. Ashburner M, Ball CA, Blake JA, Botstein D, Butler H, Cherry JM, Davis AP, Dolinski K, Dwight SS, Eppig JT, Harris MA, Hill DP, Issel-Tarver L, Kasarskis A, Lewis S, Matese JC, Richardson JE, Ringwald M, Rubin GM, Sherlock G. Gene ontology: Tool for the unification of biology. Nat. Genet. NIH Public Access; 2000. p. 25–29.

3. Carbon S, Douglass E, Dunn N, Good B, Harris NL, Lewis SE, Mungall CJ, Basu S, Chisholm RL, Dodson RJ, Hartline E, Fey P, Thomas PD, Albou LP, Ebert D, Kesling MJ, Mi H, Muruganujan A, Huang X, Poudel S, Mushayahama T, Hu JC, LaBonte SA, Siegele DA, Antonazzo G, Attrill H, Brown NH, Fexova S, Garapati P, Jones TEM, et al. The Gene Ontology Resource: 20 years and still GOing strong. *Nucleic Acids Res* Oxford University Press; 2019;**47**:D330–D338.

4. Foraita R, Spallek J, Zeeb H. Directed acyclic graphs. *Handbook of Epidemiology: Second Edition* Springer New York; 2014. p. 1481–1517.

5. Hawkins DM. The Problem of Overfitting. J. Chem. Inf. Comput. Sci. J Chem Inf Comput Sci; 2004. p. 1–12.

6. Dimensionality Reduction Techniques | Python. https://www.analyticsvidhya.com/blog/2018/08/dimensionality-reduction-techniques-python/ (15 June 2020)

7. Sorzano COS, Vargas J, Pascual-Montano A. A survey of dimensionality reduction techniques.

8. Desboulets LDD. A review on variable selection in regression analysis. Econometrics. MDPI, Open Access Journal; 2018. p. 1–27.

9. Jolliffe IT, Cadima J. Principal component analysis: A review and recent developments. Philos. Trans. R. Soc. A Math. Phys. Eng. Sci. Royal Society of London; 2016.

10. Jaadi Z. A Step by Step Explanation of Principal Component Analysis. *Towar Data Sci* 2019;1–11.

Supplementary Figures and Tables

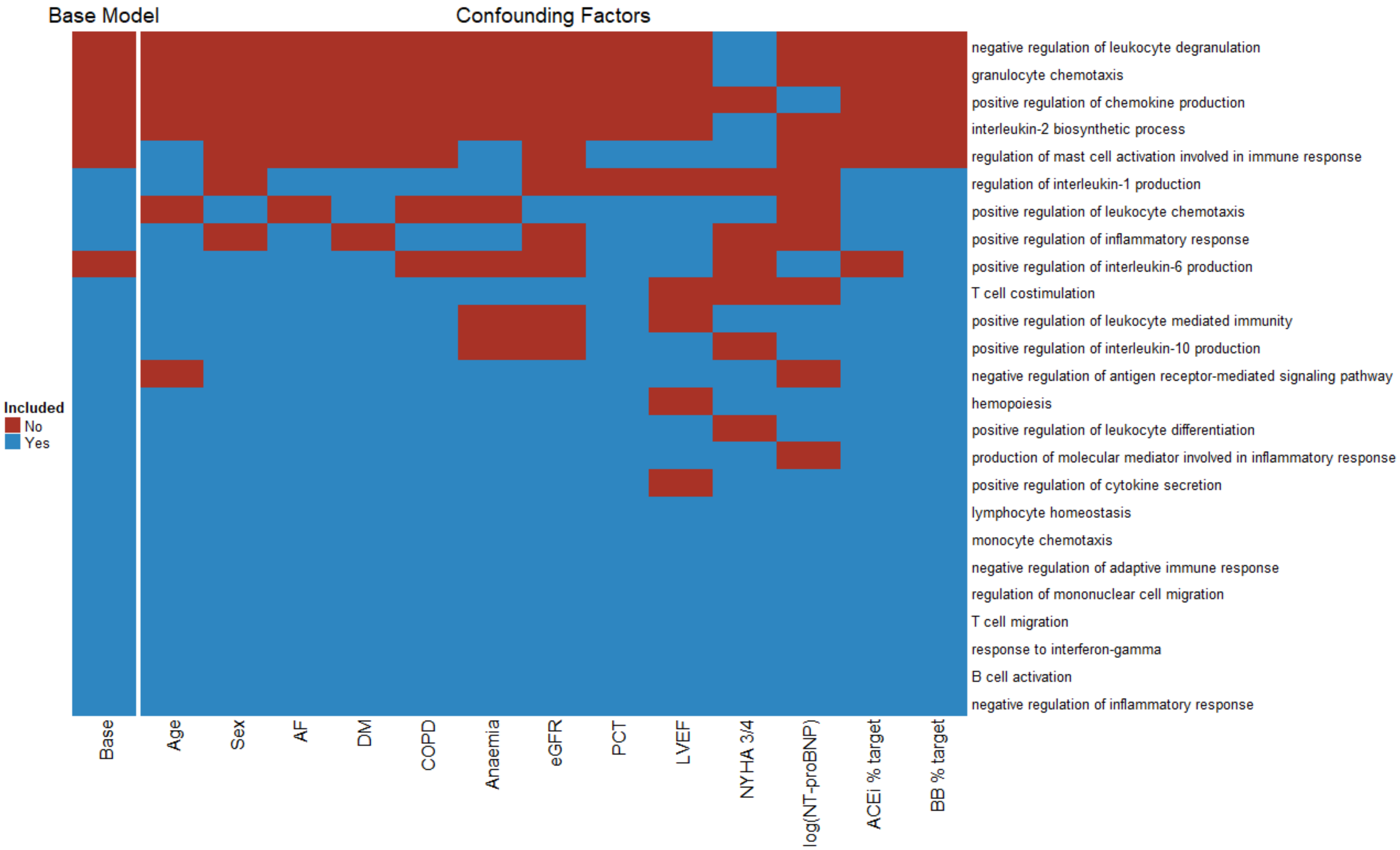

Supplementary Figure 1. Sensitivity analyses for various confounding factors, applied individually as corrections to the multivariable Cox regression model with the 64 examined GO biological processes (corrected for antibiotic use in all cases). These analyses were performed in 2022 patients with heart failure from the BIOSTAT-CHF index cohort. Processes in blue were significant and processes in red were not. Five processes that were not in the base model were found to be significant when correcting for some confounders. ACEi/BB % target refers to % of guideline-recommended dosage used at baseline. ACEi angiotensin converting enzyme inhibitor; AF atrial fibrillation; BB β-adrenoreceptor blocker; COPD chronic obstructive pulmonary disease; DM diabetes mellitus; eGFR estimated glomerular filtration ratel; GO gene ontology; PCT procalcitonin; NYHA New York heart association functional class.

All-Cause Mortality Censored at 2-year Follow-up

GO Biological Processes	HR	95% CI	p-value
positive regulation of cytokine secretion	0.44	(0.32 , 0.62)	<0.0001
positive regulation of chemokine production	0.53	(0.33 , 0.84)	0.0071
positive regulation of immunoglobulin production	0.56	(0.35 , 0.88)	0.0119
positive regulation of interferon-gamma production	0.58	(0.4 , 0.86)	0.0068
positive regulation of inflammatory response	0.69	(0.55 , 0.86)	0.0012
somatic recombination of immunoglobulin superfamily domains	0.70	(0.49 , 0.99)	0.0456
regulation of myeloid cell differentiation	0.75	(0.59 , 0.95)	0.0189
T cell costimulation	1.34	(1.01 , 1.77)	0.0427
regulation of natural killer cell mediated immunity	1.36	(1.01 , 1.83)	0.044
microglial cell activation	1.42	(1.07 , 1.88)	0.0153
production of molecular mediator involved in inflammatory response	1.48	(1.19 , 1.86)	<0.0001
positive regulation of cytokine biosynthetic process	1.48	(1.08 , 2.03)	0.0138
regulation of interleukin-12 production	1.61	(1.14 , 2.28)	0.0074
negative regulation of adaptive immune response	1.73	(1.24 , 2.41)	0.0013
positive regulation of leukocyte differentiation	2.62	(1.46 , 4.71)	0.0012

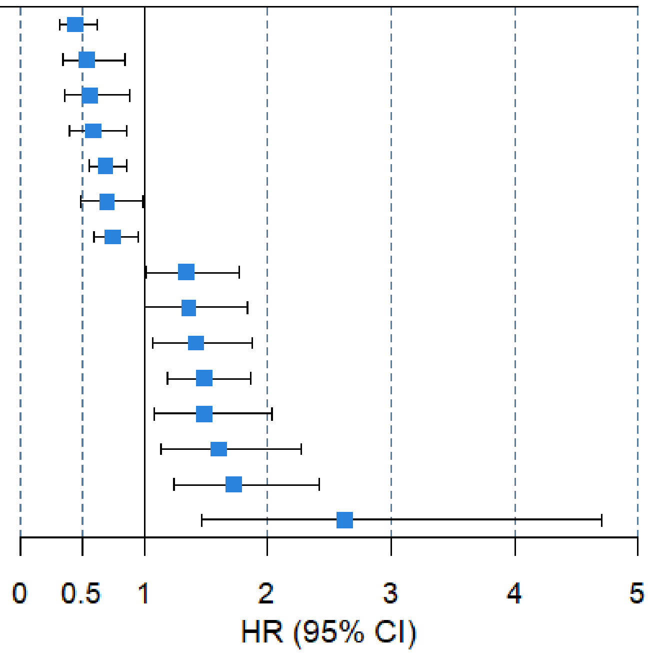

Supplementary Figure 2. Validation of multivariable Cox regression analysis of weighted scores in 1691 patients with heart failure from the independent BIOSTAT-CHF validation cohort. The outcome all-cause mortality at 2-year follow-up. Only the 15 of the 64 total processes that were significant are presented. The process *somatic recombination of immunoglobulin superfamily domains* is a truncated version of the original complete name *adaptive immune response based on somatic recombination of immune receptors built from immunoglobulin superfamily domains*.

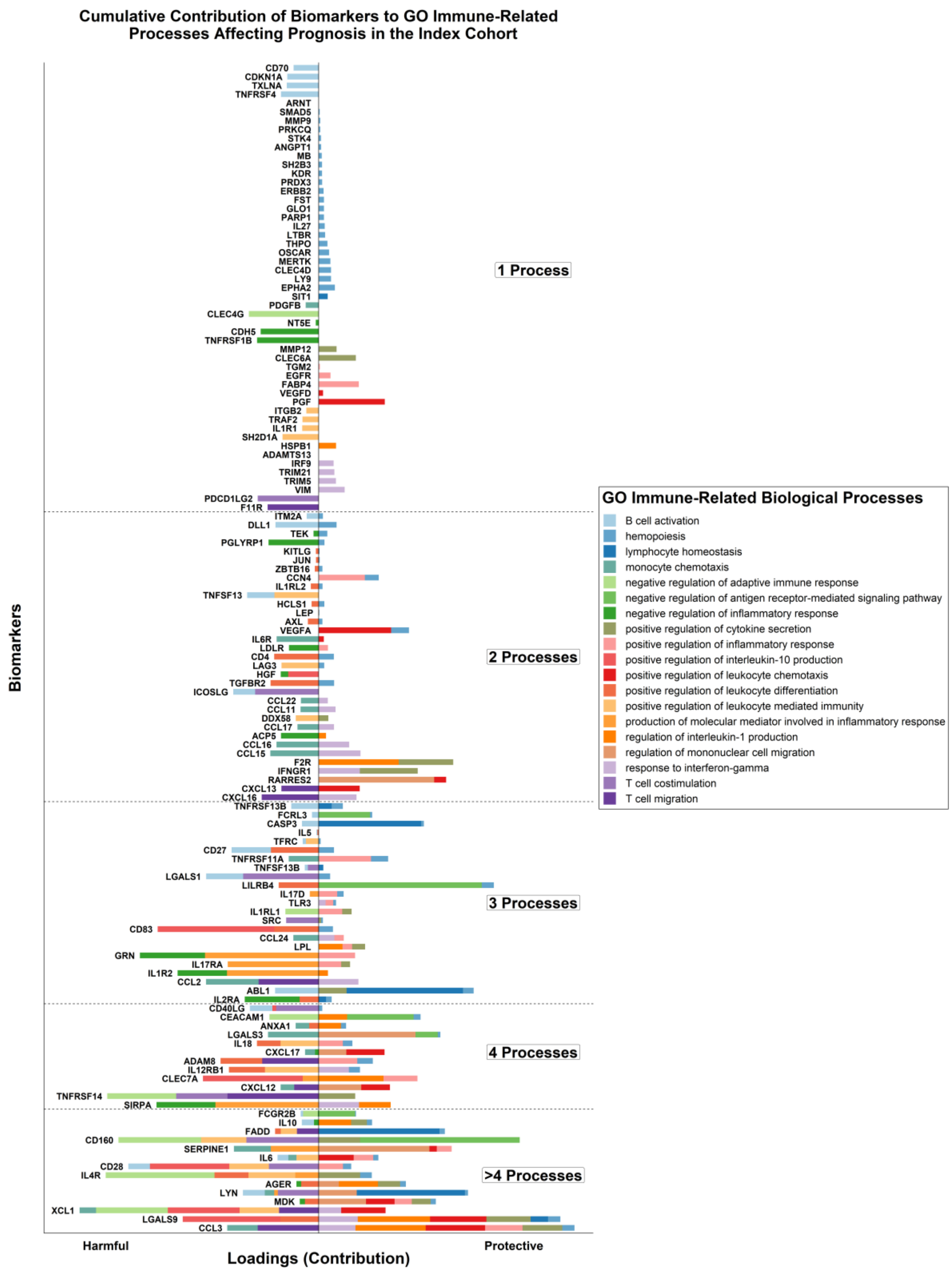

Supplementary Figure 3. Loadings (contribution) of each biomarker to the weighted scores (principal components) of processes independently associated with all-cause mortality in 2022 patients with heart failure from the BIOSTAT-CHF index cohort, sorted by the number of processes they are involved in. Biomarkers contributing to protective processes have contributions pointing to the right side of the graph and those contributing to harmful processes have contributions pointing to the left side of the graph. The two dashed lines delineate sequentially the end of biomarkers contributing only to one process and to two processes respectively. GO gene ontology

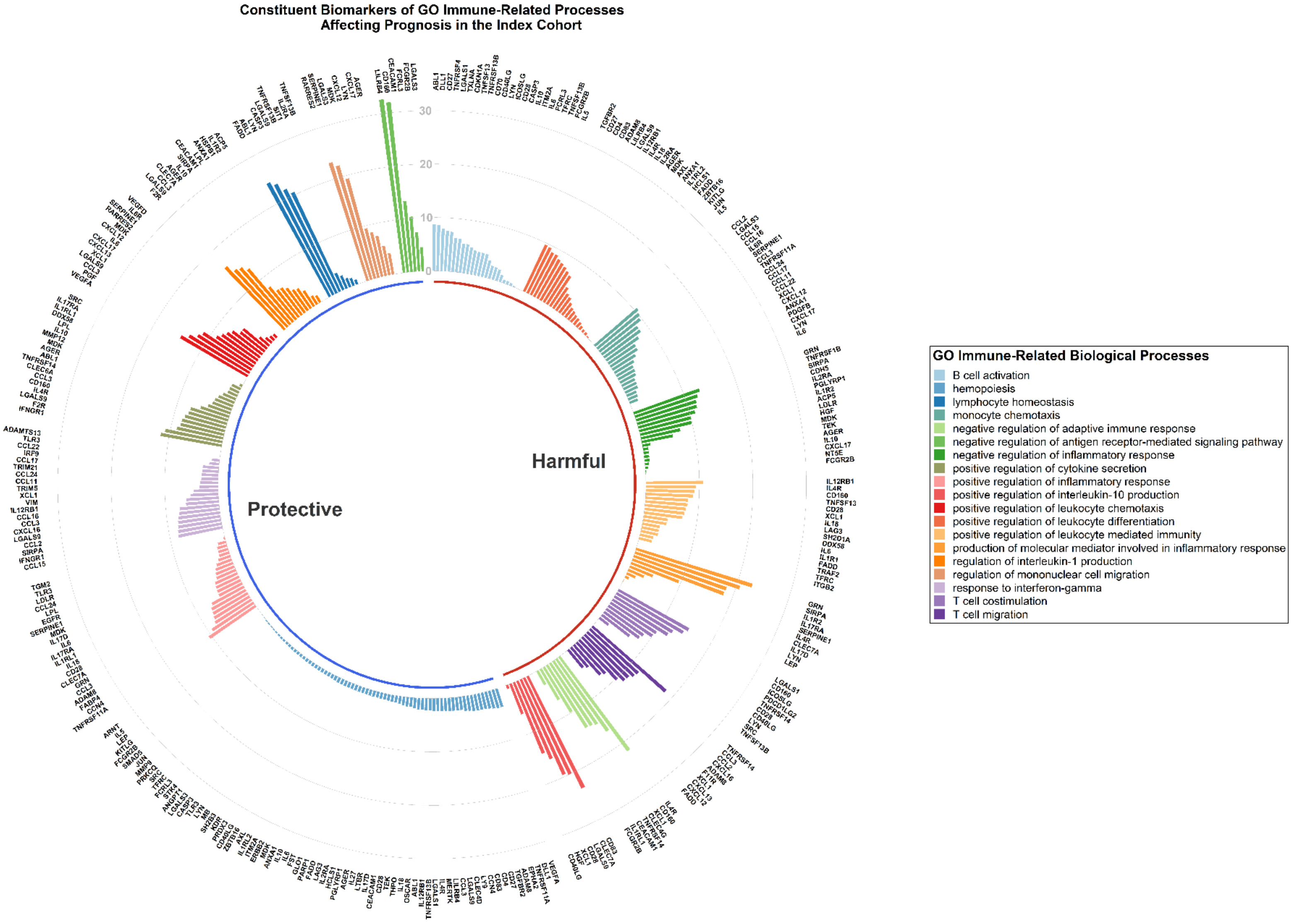

Supplementary Figure 4. Circular bar plot displaying the contribution of individual constituent biomarkers to their respective processes in 2022 patients with heart failure from the BIOSTAT-CHF index cohort, as presented in **Supplementary Figure 3**, but also grouped by process and separated into protective and harmful categories. GO gene ontology

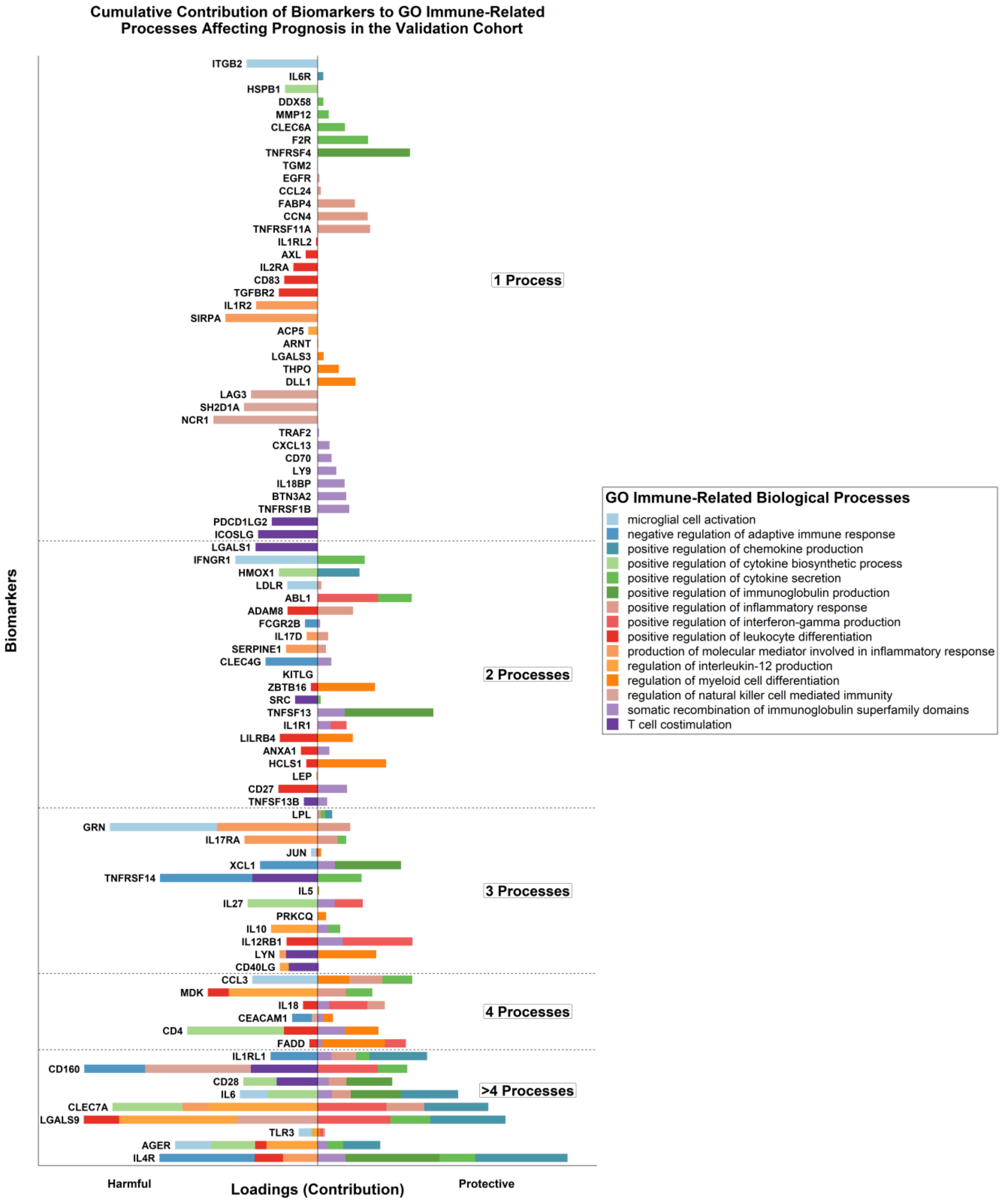

Supplementary Figure 5. Loadings (contribution) of each biomarker to the weighted scores (principal components) of processes independently associated with all-cause mortality in 1691 patients from the independent BIOSTAT-CHF validation cohort, sorted by the number of processes they are involved in. Biomarkers contributing to protective processes have contributions pointing to the right side of the graph and those contributing to harmful processes have contributions pointing to the left side of the graph. The two dashed lines delineate sequentially the end of biomarkers contributing only to one process and to two processes respectively. GO gene ontology

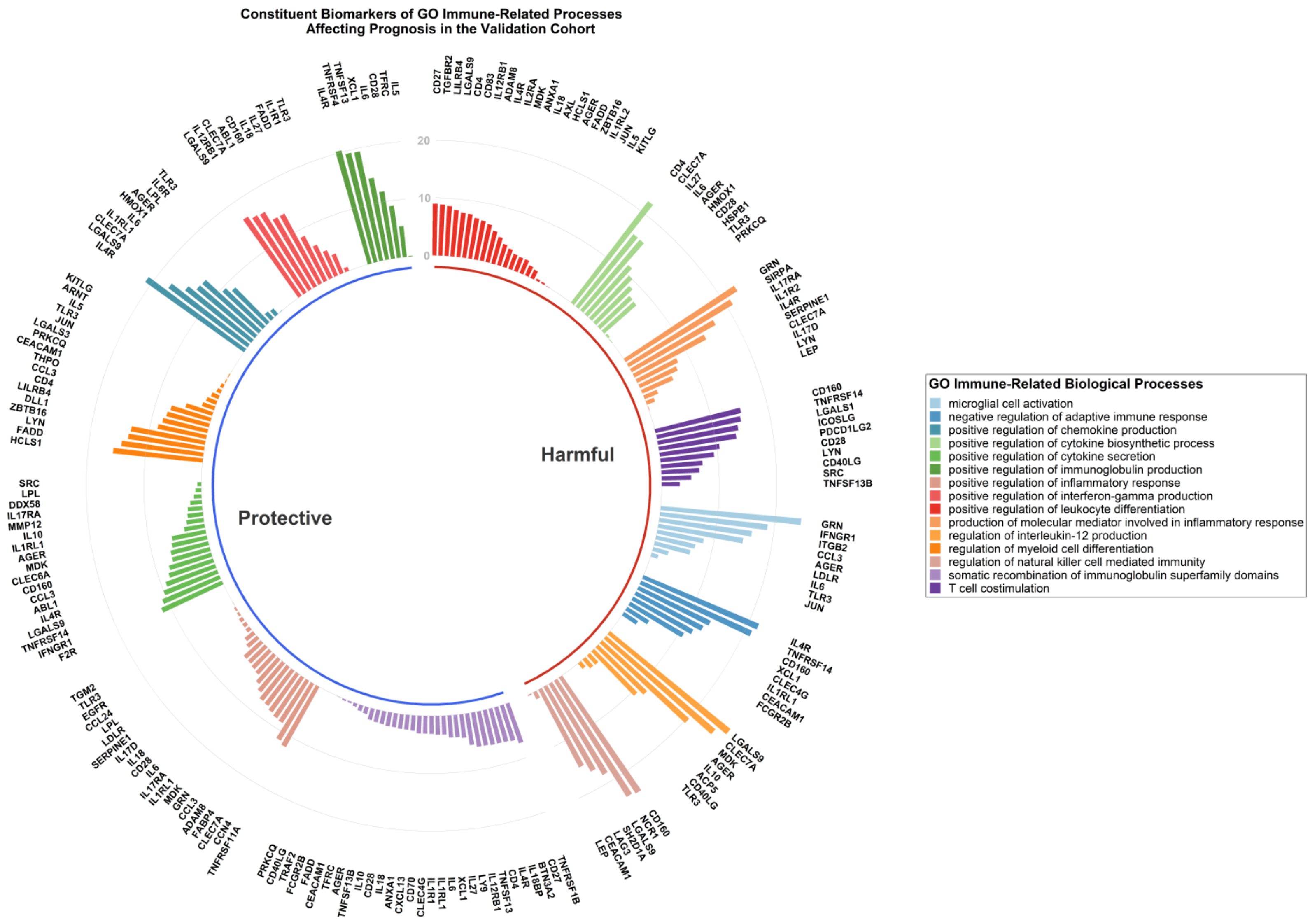

Supplementary Figure 6. Circular bar plot displaying the contribution of individual constituent biomarkers to their respective processes in 1691 patients with heart failure from the BIOSTAT-CHF validation cohort, as presented in **Supplementary Figure 5**, but also grouped by process and separated into protective and harmful categories. GO gene ontology

Supplementary Table 1. A complete list of the 92 biomarkers measured as part of the CVD-II biomarker panel. The hGNC abbreviation, Uniprot identifier and complete name for each biomarker are listed. **biomarker selected based on pathway overrepresentation analysis; hGNC human genome organization gene nomenclature committee

hGNC Abbreviation	Uniprot Identifier	Complete Name
**ACE2**	Q9BYF1	angiotensin I converting enzyme 2
**ADAMTS13 ****	Q76LX8	ADAM metallopeptidase with thrombospondin type 1 motif 13
**ADM**	P35318	adrenomedullin
**AGER ****	Q15109	advanced glycosylation end-product specific receptor
**AGRP**	O00253	agouti related neuropeptide
**AMBP**	P02760	alpha-1-microglobulin/bikunin precursor
**ANGPT1 ****	Q15389	angiopoietin 1
**BMP6**	P22004	bone morphogenetic protein 6
**BOC**	Q9BWV1	BOC cell adhesion associated, oncogene regulated
**CA5A**	P35218	carbonic anhydrase 5A
**CBLIF**	P27352	cobalamin binding intrinsic factor
**CCL17 ****	Q92583	C-C motif chemokine ligand 17
**CCL3 ****	P10147	C-C motif chemokine ligand 3
**CD4 ****	P01730	CD4 molecule
**CD40LG ****	P29965	CD40 ligand
**CD84 ****	Q9UIB8	CD84 molecule
**CEACAM8 ****	P31997	CEA cell adhesion molecule 8
**CTRC**	Q99895	chymotrypsin C
**CTSL ****	P07711	cathepsin L
**CXCL1 ****	P09341	C-X-C motif chemokine ligand 1
**DCN**	P07585	decorin
**DECR1**	Q16698	2,4-dienoyl-CoA reductase 1
**DKK1**	O94907	dickkopf WNT signaling pathway inhibitor 1
**F2R ****	P25116	coagulation factor II thrombin receptor
**F3**	P13726	coagulation factor III, tissue factor
**FABP2**	P12104	fatty acid binding protein 2
**FABP6**	P51161	fatty acid binding protein 6
**FCGR2B ****	P31994	Fc fragment of IgG receptor IIb
**FGF21**	Q9NSA1	fibroblast growth factor 21
**FGF23**	Q9GZV9	fibroblast growth factor 23
**FST ****	P19883	follistatin
**GDF2 ****	Q9UK05	growth differentiation factor 2
**GH1**	P01241	growth hormone 1
**GLO1 ****	Q04760	glyoxalase I
**HAO1**	Q9UJM8	hydroxyacid oxidase 1
**HAVCR1**	Q96D42	hepatitis A virus cellular receptor 1
**HBEGF**	Q99075	heparin binding EGF like growth factor
**HMOX1 ****	P09601	heme oxygenase 1
**HSPB1 ****	P04792	heat shock protein family B (small) member 1
**IDUA**	P35475	alpha-L-iduronidase
**IKBKG ****	Q9Y6K9	inhibitor of nuclear factor kappa B kinase regulatory subunit gamma
**IL16**	Q14005	interleukin 16
**IL17D ****	Q8TAD2	interleukin 17D
**IL18 ****	Q14116	interleukin 18
**IL1RL2 ****	Q9HB29	interleukin 1 receptor like 2
**IL1RN ****	P18510	interleukin 1 receptor antagonist
**IL27 ****	Q8NEV9	interleukin 27
**IL4R ****	P24394	interleukin 4 receptor
**IL6 ****	P05231	interleukin 6
**ITGB1BP2**	Q9UKP3	integrin subunit beta 1 binding protein 2
**KITLG ****	P21583	KIT ligand
**LEP ****	P41159	leptin
**LGALS9 ****	O00182	galectin 9

**LPL ****	P06858	lipoprotein lipase
**MARCO ****	Q9UEW3	macrophage receptor with collagenous structure
**MERTK ****	Q12866	MER proto-oncogene, tyrosine kinase
**MMP12 ****	P39900	matrix metallopeptidase 12
**MMP7**	P09237	matrix metallopeptidase 7
**NPPB**	P16860	natriuretic peptide B
**OLR1 ****	P78380	oxidized low density lipoprotein receptor 1
**OSCAR ****	Q8IYS5	osteoclast associated Ig-like receptor
**PAPPA**	Q13219	pappalysin 1
**PARP1 ****	P09874	poly(ADP-ribose) polymerase 1
**PDCD1LG2 ****	Q9BQ51	programmed cell death 1 ligand 2
**PDGFB ****	P01127	platelet derived growth factor subunit B
**PGF ****	P49763	placental growth factor
**PIGR ****	P01833	polymeric immunoglobulin receptor
**PRELP**	P51888	proline and arginine rich end leucine rich repeat protein
**PRSS27**	Q9BQR3	serine protease 27
**PRSS8**	Q16651	serine protease 8
**PTX3 ****	P26022	pentraxin 3
**REN**	P00797	renin
**SELPLG**	Q14242	selectin P ligand
**SERPINA12**	Q8IW75	serpin family A member 12
**SLAMF7 ****	Q9NQ25	SLAM family member 7
**SOD2**	P04179	superoxide dismutase 2
**SORT1**	Q99523	sortilin 1
**SPON2 ****	Q9BUD6	spondin 2
**SRC ****	P12931	SRC proto-oncogene, non-receptor tyrosine kinase
**STK4 ****	Q13043	serine/threonine kinase 4
**TEK ****	Q02763	TEK receptor tyrosine kinase
**TGM2 ****	P21980	transglutaminase 2
**THBD**	P07204	thrombomodulin
**THBS2**	P35442	thrombospondin 2
**THPO ****	P40225	thrombopoietin
**TNFRSF10A**	O00220	TNF receptor superfamily member 10a
**TNFRSF10B**	O14763	TNF receptor superfamily member 10b
**TNFRSF11A ****	Q9Y6Q6	TNF receptor superfamily member 11a
**TNFRSF13B ****	O14836	TNF receptor superfamily member 13B
**VEGFD ****	O43915	vascular endothelial growth factor D
**VSIG2**	Q96IQ7	V-set and immunoglobulin domain containing 2
**XCL1 ****	P47992	X-C motif chemokine ligand 1

Supplementary Table 2. A complete list of the 92 biomarkers measured as part of the CVD-III biomarker panel. The hGNC abbreviation, Uniprot identifier and complete name for each biomarker are listed. *excluded due to >10% of measurements being under the assay lower limit of detection; **biomarker selected based on pathway overrepresentation analysis; hGNC human genome organization gene nomenclature committee

hGNC Abbreviation	Uniprot Identifier	Complete Name
**ACP5 ****	P13686	acid phosphatase 5, tartrate resistant
**ALCAM**	Q13740	activated leukocyte cell adhesion molecule
**ANPEP ****	P15144	alanyl aminopeptidase, membrane
**AXL ****	P30530	AXL receptor tyrosine kinase
**AZU1 ***	P20160	azurocidin 1
**BLMH ***	Q13867	bleomycin hydrolase
**CASP3 ****	P42574	caspase 3
**CCL15 ****	Q16663	C-C motif chemokine ligand 15
**CCL16 ****	O15467	C-C motif chemokine ligand 16
**CCL2 ****	P13500	C-C motif chemokine ligand 2
**CCL22 ****	O00626	C-C motif chemokine ligand 22
**CCL24 ****	O00175	C-C motif chemokine ligand 24
**CD163**	Q86VB7	CD163 molecule
**CD93 ****	Q9NPY3	CD93 molecule
**CDH5 ****	P33151	cadherin 5
**CHI3L1 ****	P36222	chitinase 3 like 1
**CHIT1 ****	Q13231	chitinase 1
**CNTN1**	Q12860	contactin 1
**COL1A1**	P02452	collagen type I alpha 1 chain
**CPA1**	P15085	carboxypeptidase A1
**CPB1**	P15086	carboxypeptidase B1
**CSTB ***	P04080	cystatin B
**CTSD ****	P07339	cathepsin D
**CTSZ ****	Q9UBR2	cathepsin Z
**CXCL16 ****	Q9H2A7	C-X-C motif chemokine ligand 16
**DLK1**	P80370	delta like non-canonical Notch ligand 1
**EGFR ****	P00533	epidermal growth factor receptor
**EPCAM**	P16422	epithelial cell adhesion molecule
**EPHB4**	P54760	EPH receptor B4
**F11R ****	Q9Y624	F11 receptor
**FABP4 ****	P15090	fatty acid binding protein 4
**FAS**	P25445	Fas cell surface death receptor
**GDF15**	Q99988	growth differentiation factor 15
**GRN ****	P28799	granulin precursor
**HSPG2**	P98160	heparan sulfate proteoglycan 2
**ICAM2 ****	P13598	intercellular adhesion molecule 2
**IGFBP1**	P08833	insulin like growth factor binding protein 1
**IGFBP2 ****	P18065	insulin like growth factor binding protein 2
**IGFBP7**	Q16270	insulin like growth factor binding protein 7
**IL17RA ****	Q96F46	interleukin 17 receptor A
**IL18BP ****	O95998	interleukin 18 binding protein
**IL1R1 ****	P14778	interleukin 1 receptor type 1
**IL1R2 ****	P27930	interleukin 1 receptor type 2
**IL1RL1 ****	Q01638	interleukin 1 receptor like 1
**IL2RA ****	P01589	interleukin 2 receptor subunit alpha
**IL6R ****	P08887	interleukin 6 receptor
**ITGB2 ****	P05107	integrin subunit beta 2
**KLK6 ***	Q92876	kallikrein related peptidase 6
**LDLR ****	P01130	low density lipoprotein receptor
**LGALS3 ****	P17931	galectin 3
**LGALS4**	P56470	galectin 4
**LTBR ****	P36941	lymphotoxin beta receptor

**MB ****	P02144	myoglobin
**MEPE**	Q9NQ76	matrix extracellular phosphoglycoprotein
**MMP2**	P08253	matrix metallopeptidase 2
**MMP3 ****	P08254	matrix metallopeptidase 3
**MMP9 ****	P14780	matrix metallopeptidase 9
**MPO ****	P05164	myeloperoxidase
**NOTCH3**	Q9UM47	notch receptor 3
**NPPB**	P16860	natriuretic peptide B
**PCSK9**	Q8NBP7	proprotein convertase subtilisin/kexin type 9
**PDGFA**	P04085	platelet derived growth factor subunit A
**PECAM1 ****	P16284	platelet and endothelial cell adhesion molecule 1
**PGLYRP1 ****	O75594	peptidoglycan recognition protein 1
**PI3 ***	P19957	peptidase inhibitor 3
**PLAT**	P00750	plasminogen activator, tissue type
**PLAU ****	P00749	plasminogen activator, urokinase
**PLAUR ****	Q03405	plasminogen activator, urokinase receptor
**PON3**	Q15166	paraoxonase 3
**PRTN3 ***	P24158	proteinase 3
**RARRES2 ****	Q99969	retinoic acid receptor responder 2
**RETN ****	Q9HD89	resistin
**SCGB3A2**	Q96PL1	secretoglobin family 3A member 2
**SELE**	P16581	selectin E
**SELP**	P16109	selectin P
**SERPINE1 ****	P05121	serpin family E member 1
**SFTPD ***	P35247	surfactant protein D
**SIRPA ****	P78324	signal regulatory protein alpha
**SPON1**	Q9HCB6	spondin 1
**SPP1**	P10451	secreted phosphoprotein 1
**TFF3**	Q07654	trefoil factor 3
**TFPI2**	P48307	tissue factor pathway inhibitor 2
**TFRC ****	P02786	transferrin receptor
**TIMP4**	Q99727	TIMP metallopeptidase inhibitor 4
**TNFRSF10C**	O14798	TNF receptor superfamily member 10c
**TNFRSF11B**	O00300	TNF receptor superfamily member 11b
**TNFRSF14 ****	Q92956	TNF receptor superfamily member 14
**TNFRSF1A ***	P19438	TNF receptor superfamily member 1A
**TNFRSF1B ****	P20333	TNF receptor superfamily member 1B
**TNFSF13B ****	Q9Y275	TNF superfamily member 13b
**TREML2 ****	Q5T2D2	triggering receptor expressed on myeloid cells like 2
**VWF**	P04275	von Willebrand factor

Supplementary Table 3. A complete list of the 92 biomarkers measured as part of the immune biomarker panel. The hGNC abbreviation, Uniprot identifier and complete name for each biomarker are listed. **biomarker selected based on pathway overrepresentation analysis; hGNC human genome organization gene nomenclature committee

hGNC Abbreviation	Uniprot Identifier	Complete Name
**AREG**	P15514	amphiregulin
**ARNT ****	P27540	aryl hydrocarbon receptor nuclear translocator
**BACH1**	O14867	BTB domain and CNC homolog 1
**BIRC2 ****	Q13490	baculoviral IAP repeat containing 2
**BTN3A2 ****	P78410	butyrophilin subfamily 3 member A2
**CCL11 ****	P51671	C-C motif chemokine ligand 11
**CD28 ****	P10747	CD28 molecule
**CD83 ****	Q01151	CD83 molecule
**CDSN**	Q15517	corneodesmosin
**CKAP4 ****	Q07065	cytoskeleton associated protein 4
**CLEC4A ****	Q9UMR7	C-type lectin domain family 4 member A
**CLEC4C ****	Q8WTT0	C-type lectin domain family 4 member C
**CLEC4D ****	Q8WXI8	C-type lectin domain family 4 member D
**CLEC4G ****	Q6UXB4	C-type lectin domain family 4 member G
**CLEC6A ****	Q6EIG7	C-type lectin domain containing 6A
**CLEC7A ****	Q9BXN2	C-type lectin domain containing 7A
**CNTNAP2**	Q9UHC6	contactin associated protein like 2
**CXADR ****	P78310	CXADR Ig-like cell adhesion molecule
**CXCL12 ****	P48061	C-X-C motif chemokine ligand 12
**DAPP1**	Q9UN19	dual adaptor of phosphotyrosine and 3-phosphoinositides 1
**DCBLD2**	Q96PD2	discoidin, CUB and LCCL domain containing 2
**DCTN1**	Q14203	dynactin subunit 1
**DDX58 ****	O95786	DExD/H-box helicase 58
**DFFA**	O00273	DNA fragmentation factor subunit alpha
**DGKZ**	Q13574	diacylglycerol kinase zeta
**DPP10**	Q8N608	dipeptidyl peptidase like 10
**EDAR**	Q9UNE0	ectodysplasin A receptor
**EGLN1**	Q9GZT9	egl-9 family hypoxia inducible factor 1
**EIF4G1**	Q04637	eukaryotic translation initiation factor 4 gamma 1
**EIF5A**	P63241	eukaryotic translation initiation factor 5A
**FAM3B**	P58499	family with sequence similarity 3 member B
**FCRL3 ****	Q96P31	Fc receptor like 3
**FCRL6**	Q6DN72	Fc receptor like 6
**FGF2**	P09038	fibroblast growth factor 2
**FXYD5**	Q96DB9	FXYD domain containing ion transport regulator 5
**GALNT3**	Q14435	polypeptide N-acetylgalactosaminyltransferase 3
**GLB1 ****	P16278	galactosidase beta 1
**HCLS1 ****	P14317	hematopoietic cell-specific Lyn substrate 1
**HEXIM1 ****	O94992	HEXIM P-TEFb complex subunit 1
**HNMT**	P50135	histamine N-methyltransferase
**HSD11B1**	P28845	hydroxysteroid 11-beta dehydrogenase 1
**ICA1**	Q05084	islet cell autoantigen 1
**IFNLR1**	Q8IU57	interferon lambda receptor 1
**IL10 ****	P22301	interleukin 10
**IL12RB1 ****	P42701	interleukin 12 receptor subunit beta 1
**IL5 ****	P05113	interleukin 5
**IL6 ****	P05231	interleukin 6
**IRAK1 ****	P51617	interleukin 1 receptor associated kinase 1
**IRAK4 ****	Q9NWZ3	interleukin 1 receptor associated kinase 4
**IRF9 ****	Q00978	interferon regulatory factor 9
**ITGA11**	Q9UKX5	integrin subunit alpha 11
**ITGA6**	P23229	integrin subunit alpha 6
**ITGB6**	P18564	integrin subunit beta 6

**ITM2A** **	O43736	integral membrane protein 2A
**JUN** **	P05412	Jun proto-oncogene, AP-1 transcription factor subunit
**KLRD1** **	Q13241	killer cell lectin like receptor D1
**KPNA1**	P52294	karyopherin subunit alpha 1
**KRT19**	P08727	keratin 19
**LAG3** **	P18627	lymphocyte activating 3
**LAMP3**	Q9UQV4	lysosomal associated membrane protein 3
**LILRB4** **	Q8NHJ6	leukocyte immunoglobulin like receptor B4
**LY75**	O60449	lymphocyte antigen 75
**MASP1**	P48740	mannan binding lectin serine peptidase 1
**MGMT**	P16455	O-6-methylguanine-DNA methyltransferase
**MILR1** **	Q7Z6M3	mast cell immunoglobulin like receptor 1
**NCR1** **	O76036	natural cytotoxicity triggering receptor 1
**NF2**	P35240	neurofibromin 2
**NFATC3** **	Q12968	nuclear factor of activated T cells 3
**NTF4**	P34130	neurotrophin 4
**PADI2** **	Q9Y2J8	peptidyl arginine deiminase 2
**PIK3AP1** **	Q6ZUJ8	phosphoinositide-3-kinase adaptor protein 1
**PLXNA4**	Q9HCM2	plexin A4
**PPP1R9B**	Q96SB3	protein phosphatase 1 regulatory subunit 9B
**PRDX1** **	Q06830	peroxiredoxin 1
**PRDX3** **	P30048	peroxiredoxin 3
**PRDX5**	P30044	peroxiredoxin 5
**PRKCQ** **	Q04759	protein kinase C theta
**PSIP1**	O75475	PC4 and SFRS1 interacting protein 1
**PTH1R**	Q03431	parathyroid hormone 1 receptor
**SH2B3** **	Q9UQQ2	SH2B adaptor protein 3
**SH2D1A** **	O60880	SH2 domain containing 1A
**SIT1** **	Q9Y3P8	signaling threshold regulating transmembrane adaptor 1
**SPRY2**	O43597	sprouty RTK signaling antagonist 2
**SRPK2**	P78362	SRSF protein kinase 2
**STC1**	P52823	stanniocalcin 1
**TANK** **	Q92844	TRAF family member associated NFKB activator
**TPSAB1**	Q15661	tryptase alpha/beta 1
**TRAF2** **	Q12933	TNF receptor associated factor 2
**TREM1**	Q9NP99	triggering receptor expressed on myeloid cells 1
**TRIM21** **	P19474	tripartite motif containing 21
**TRIM5** **	Q9C035	tripartite motif containing 5
**ZBTB16** **	Q05516	zinc finger and BTB domain containing 16

Supplementary Table 4. A complete list of the 92 biomarkers measured as part of the oncology biomarker panel. The hGNC abbreviation, Uniprot identifier and complete name for each biomarker are listed. **biomarker selected based on pathway overrepresentation analysis

hGNC Abbreviation	Uniprot Identifier	Complete Name
**ABL1 ** **	P00519	ABL proto-oncogene 1, non-receptor tyrosine kinase
**ADAM8 ** **	P78325	ADAM metallopeptidase domain 8
**ADAMTS15**	Q8TE58	ADAM metallopeptidase with thrombospondin type 1 motif 15
**ANXA1 ** **	P04083	annexin A1
**AREG**	P15514	amphiregulin
**CA9**	Q16790	carbonic anhydrase 9
**CCN1**	O00622	cellular communication network factor 1
**CCN4 ** **	O95388	cellular communication network factor 4
**CD160 ** **	O95971	CD160 molecule
**CD207**	Q9UJ71	CD207 molecule
**CD27 ** **	P26842	CD27 molecule
**CD48**	P09326	CD48 molecule
**CD70 ** **	P32970	CD70 molecule
**CDKN1A ** **	P38936	cyclin dependent kinase inhibitor 1A
**CEACAM1 ** **	P13688	CEA cell adhesion molecule 1
**CEACAM5**	P06731	CEA cell adhesion molecule 5
**CPE**	P16870	carboxypeptidase E
**CRNN**	Q9UBG3	cornulin
**CTSV**	O60911	cathepsin V
**CXCL13 ** **	O43927	C-X-C motif chemokine ligand 13
**CXCL17 ** **	Q6UXB2	C-X-C motif chemokine ligand 17
**DLL1 ** **	O00548	delta like canonical Notch ligand 1
**EGF**	P01133	epidermal growth factor
**EPHA2 ** **	P29317	EPH receptor A2
**ERBB2 ** **	P04626	erb-b2 receptor tyrosine kinase 2
**ERBB3**	P21860	erb-b2 receptor tyrosine kinase 3
**ERBB4**	Q15303	erb-b2 receptor tyrosine kinase 4
**ESM1**	Q9NQ30	endothelial cell specific molecule 1
**FADD ** **	Q13158	Fas associated via death domain
**FASLG**	P48023	Fas ligand
**FCRLB**	Q6BAA4	Fc receptor like B
**FGFBP1**	Q14512	fibroblast growth factor binding protein 1
**FLT4**	P35916	fms related tyrosine kinase 4
**FOLR1**	P15328	folate receptor alpha
**FOLR3 ** **	P41439	folate receptor gamma
**FURIN**	P09958	furin, paired basic amino acid cleaving enzyme
**GPC1**	P35052	glypican 1
**GPNMB ** **	Q14956	glycoprotein nmb
**GZMB ** **	P10144	granzyme B
**GZMH**	P20718	granzyme H
**HGF ** **	P14210	hepatocyte growth factor
**ICOSLG ** **	O75144	inducible T cell costimulator ligand
**IFNGR1 ** **	P15260	interferon gamma receptor 1
**IGF1R**	P08069	insulin like growth factor 1 receptor
**IL6 ** **	P05231	interleukin 6
**ITGAV ** **	P06756	integrin subunit alpha V
**ITGB5**	P18084	integrin subunit beta 5
**KDR ** **	P35968	kinase insert domain receptor
**KITLG ** **	P21583	KIT ligand
**KLK11**	Q9UBX7	kallikrein related peptidase 11
**KLK13**	Q9UKR3	kallikrein related peptidase 13
**KLK14**	Q9P0G3	kallikrein related peptidase 14
**KLK8**	O60259	kallikrein related peptidase 8

**LGALS1 ****	P09382	galectin 1
**LY9 ****	Q9HBG7	lymphocyte antigen 9
**LYN ****	P07948	LYN proto-oncogene, Src family tyrosine kinase
**LYPD3**	O95274	LY6/PLAUR domain containing 3
**MDK ****	P21741	midkine
**METAP2**	P50579	methionyl aminopeptidase 2
**MIA**	Q16674	MIA SH3 domain containing
**MICA**	Q29983	MHC class I polypeptide-related sequence A
**MSLN**	Q13421	mesothelin
**MUC16 ****	Q8WXI7	mucin 16, cell surface associated
**NECTIN4**	Q96NY8	nectin cell adhesion molecule 4
**NT5E ****	P21589	5'-nucleotidase ecto
**PODXL**	O00592	podocalyxin like
**PPY**	P01298	pancreatic polypeptide
**RET**	P07949	ret proto-oncogene
**RSPO3**	Q9BXY4	R-spondin 3
**S100A11 ****	P31949	S100 calcium binding protein A11
**S100A4**	P26447	S100 calcium binding protein A4
**SCAMP3**	O14828	secretory carrier membrane protein 3
**SDC1**	P18827	syndecan 1
**SEZ6L**	Q9BYH1	seizure related 6 homolog like
**SMAD5 ****	Q99717	SMAD family member 5
**SPARC**	P09486	secreted protein acidic and cysteine rich
**TCL1A**	P56279	T cell leukemia/lymphoma 1A
**TFPI2**	P48307	tissue factor pathway inhibitor 2
**TGFA**	P01135	transforming growth factor alpha
**TGFBR2 ****	P37173	transforming growth factor beta receptor 2
**TLR3 ****	O15455	toll like receptor 3
**TNFRSF19**	Q9NS68	TNF receptor superfamily member 19
**TNFRSF4 ****	P43489	TNF receptor superfamily member 4
**TNFRSF6B**	O95407	TNF receptor superfamily member 6b
**TNFSF10**	P50591	TNF superfamily member 10
**TNFSF13 ****	O75888	TNF superfamily member 13
**TXLNA ****	P40222	taxilin alpha
**VEGFA ****	P15692	vascular endothelial growth factor A
**VIM ****	P08670	vimentin
**WFDC2**	Q14508	WAP four-disulfide core domain 2
**WIF1**	Q9Y5W5	WNT inhibitory factor 1
**XPNPEP2**	O43895	X-prolyl aminopeptidase 2

Supplementary Table 5. Names, identification codes and definitions of the 64 GO immune-related biological processes that were examined for their effect on all-cause mortality. Processes are split into the tree main parent processes related to the immune system. A link to the complete page for each process in the online GO annotation browser (QuickGO) is also provided. QuickGO provides additional information regarding all gene product constituents of each process and shows the spatial relationships of each process to other biological processes. GO gene ontology

	GO Process Name	GO Process ID	Definition	QuickGO Link
Immune System Process	adaptive immune response based on somatic recombination of immune receptors built from immunoglobulin superfamily domains	GO:0002460	An immune response mediated by lymphocytes expressing specific receptors for antigen produced through a somatic diversification process that includes somatic recombination of germline gene segments encoding immunoglobulin superfamily domains. Recombined receptors for antigen encoded by immunoglobulin superfamily domains include T cell receptors and immunoglobulins (antibodies) produced by B cells. The first encounter with antigen elicits a primary immune response that is slow and not of great magnitude. T and B cells selected by antigen become activated and undergo clonal expansion. A fraction of antigen-reactive T and B cells become memory cells, whereas others differentiate into effector cells. The memory cells generated during the primary response enable a much faster and stronger secondary immune response upon subsequent exposures to the same antigen (immunological memory). An example of this is the adaptive immune response found in Mus musculus.	
	B cell activation	GO:0042113	The change in morphology and behavior of a mature or immature B cell resulting from exposure to a mitogen, cytokine, chemokine, cellular ligand, or an antigen for which it is specific.	
	granulocyte activation	GO:0036230	The change in morphology and behavior of a granulocyte resulting from exposure to a cytokine, chemokine, cellular ligand, or soluble factor.	
	hemopoiesis	GO:0030097	The process whose specific outcome is the progression of the myeloid and lymphoid derived organ/tissue systems of the blood and other parts of the body over time, from formation to the mature structure. The site of hemopoiesis is variable during development, but occurs primarily in bone marrow or kidney in many adult vertebrates.	
	immune response-regulating signaling pathway	GO:0002764	The cascade of processes by which a signal interacts with a receptor, causing a change in the level or activity of a second messenger or other downstream target, and ultimately leading to the activation, perpetuation, or inhibition of an immune response.	
	immunoglobulin secretion	GO:0048305	The regulated release of immunoglobulins from a B cell or plasma cell, whose mechanism includes the use of alternate polyadenylylation signals to favor the biosynthesis of secreted forms of immunoglobulin over membrane-bound immunoglobulin.	
	innate immune response-activating signal transduction	GO:0002758	The cascade of processes by which a signal interacts with a receptor, causing a change in the level or activity of a second messenger or other downstream target, and ultimately leading to activation or perpetuation of an innate immune response.	
	lymphocyte activation involved in immune response	GO:0002285	A change in morphology and behavior of a lymphocyte resulting from exposure to a specific antigen, mitogen, cytokine, chemokine, cellular ligand, or soluble factor, leading to the initiation or perpetuation of an immune response.	
	lymphocyte chemotaxis	GO:0048247	The directed movement of a lymphocyte in response to an external stimulus.	
	lymphocyte differentiation	GO:0030098	The process in which a relatively unspecialized precursor cell acquires specialized features of a lymphocyte. A lymphocyte is a leukocyte commonly found in the blood and lymph that has the characteristics of a large nucleus, a neutral staining cytoplasm, and prominent heterochromatin.	
	lymphocyte homeostasis	GO:0002260	The process of regulating the proliferation and elimination of lymphocytes such that the total number of lymphocytes within a whole or part of an organism is stable over time in the absence of an outside stimulus.	
	lymphocyte proliferation	GO:0046651	The expansion of a lymphocyte population by cell division.	
	microglial cell activation	GO:0001774	The change in morphology and behavior of a microglial cell resulting from exposure to a cytokine, chemokine, cellular ligand, or soluble factor.	
	monocyte chemotaxis	GO:0002548	The movement of a monocyte in response to an external stimulus.	
	myeloid cell activation involved in immune response	GO:0002275	A change in the morphology or behavior of a myeloid cell resulting from exposure to an activating factor such as a cellular or soluble ligand, leading to the initiation or perpetuation of an immune response.	
	myeloid leukocyte mediated immunity	GO:0002444	Any process involved in the carrying out of an immune response by a myeloid leukocyte.	

	natural killer cell mediated immunity	GO:0002228	The promotion of an immune response by natural killer cells through direct recognition of target cells or through the release of cytokines.	
	negative regulation of adaptive immune response	GO:0002820	Any process that stops, prevents, or reduces the frequency, rate, or extent of an adaptive immune response.	
	negative regulation of antigen receptor-mediated signaling pathway	GO:0050858	Any process that stops, prevents, or reduces the frequency, rate or extent of signaling pathways initiated by the cross-linking of an antigen receptor on a B- or T cell.	
	negative regulation of leukocyte degranulation	GO:0043301	Any process that stops, prevents, or reduces the rate of leukocyte degranulation.	
	negative regulation of leukocyte differentiation	GO:1902106	Any process that stops, prevents or reduces the frequency, rate or extent of leukocyte differentiation.	
	negative regulation of lymphocyte activation	GO:0051250	Any process that stops, prevents, or reduces the frequency, rate or extent of lymphocyte activation.	
	neutrophil degranulation	GO:0043312	The regulated exocytosis of secretory granules containing preformed mediators such as proteases, lipases, and inflammatory mediators by a neutrophil.	
	neutrophil homeostasis	GO:0001780	The process of regulating the proliferation and elimination of neutrophils such that the total number of neutrophils within a whole or part of an organism is stable over time in the absence of an outside stimulus.	
	positive regulation of adaptive immune response	GO:0002821	Any process that activates or increases the frequency, rate, or extent of an adaptive immune response.	
	positive regulation of cellular extravasation	GO:0002693	Any process that activates or increases the frequency, rate, or extent of cellular extravasation.	
	positive regulation of cytokine production involved in immune response	GO:0002720	Any process that activates or increases the frequency, rate, or extent of cytokine production that contributes to an immune response.	
	positive regulation of immunoglobulin production	GO:0002639	Any process that activates or increases the frequency, rate, or extent of immunoglobulin production.	
	positive regulation of innate immune response	GO:0045089	Any process that activates or increases the frequency, rate or extent of the innate immune response, the organism's first line of defense against infection.	
	positive regulation of leukocyte chemotaxis	GO:0002690	Any process that activates or increases the frequency, rate, or extent of leukocyte chemotaxis.	
	positive regulation of leukocyte differentiation	GO:1902107	Any process that activates or increases the frequency, rate or extent of leukocyte differentiation.	
	positive regulation of leukocyte mediated immunity	GO:0002705	Any process that activates or increases the frequency, rate, or extent of leukocyte mediated immunity.	
	positive regulation of lymphocyte activation	GO:0051251	Any process that activates or increases the frequency, rate or extent of lymphocyte activation.	
	positive regulation of lymphocyte migration	GO:2000403	Any process that activates or increases the frequency, rate or extent of lymphocyte migration.	
	regulation of macrophage activation	GO:0043030	Any process that modulates the frequency or rate of macrophage activation.	
	regulation of mast cell activation involved in immune response	GO:0033006	Any process that modulates the frequency, rate, or extent of mast cell activation as part of an immune response.	
	regulation of mononuclear cell migration	GO:0071675	Any process that modulates the rate, frequency or extent of mononuclear cell migration. Mononuclear cell migration is the movement of a mononuclear cell within or between different tissues and organs of the body.	
	regulation of myeloid cell differentiation	GO:0045637	Any process that modulates the frequency, rate or extent of myeloid cell differentiation.	
	response to interferon-gamma	GO:0034341	Any process that results in a change in state or activity of a cell or an organism (in terms of movement, secretion, enzyme production, gene expression, etc.) as a result of an interferon-gamma stimulus. Interferon-gamma is also known as type II interferon.	
	T cell activation	GO:0042110	The change in morphology and behavior of a mature or immature T cell resulting from exposure to a mitogen, cytokine, chemokine, cellular ligand, or an antigen for which it is specific.	
	T cell costimulation	GO:0031295	The process of providing, via surface-bound receptor-ligand pairs, a second, antigen-independent, signal in addition to that provided by the T cell receptor to augment T cell activation.	

	T cell migration	GO:0072678	The movement of a T cell within or between different tissues and organs of the body.	
Defense Response	activation of innate immune response	GO:0002218	Any process that initiates an innate immune response. Innate immune responses are defense responses mediated by germline encoded components that directly recognize components of potential pathogens. Examples of this process include activation of the hypersensitive response of Arabidopsis thaliana and activation of any NOD or TLR signaling pathway in vertebrate species.	
	inflammatory response to wounding	GO:0090594	The immediate defensive reaction by vertebrate tissue to injury caused by chemical or physical agents.	
	microglial cell activation	GO:0001774	The change in morphology and behavior of a microglial cell resulting from exposure to a cytokine, chemokine, cellular ligand, or soluble factor.	
	negative regulation of inflammatory response	GO:0050728	Any process that stops, prevents, or reduces the frequency, rate or extent of the inflammatory response.	
	positive regulation of inflammatory response	GO:0050729	Any process that activates or increases the frequency, rate or extent of the inflammatory response.	
	production of molecular mediator involved in inflammatory response	GO:0002532	The synthesis or release of any molecular mediator of the inflammatory response following an inflammatory stimulus, resulting in an increase in its intracellular or extracellular levels.	
	regulation of natural killer cell mediated immunity	GO:0002715	Any process that modulates the frequency, rate, or extent of natural killer cell mediated immunity.	
	regulation of neuroinflammatory response	GO:0150077	Any process that modulates the frequency, rate or extent of neuroinflammatory response.	
	response to interferon-gamma	GO:0034341	Any process that results in a change in state or activity of a cell or an organism (in terms of movement, secretion, enzyme production, gene expression, etc.) as a result of an interferon-gamma stimulus. Interferon-gamma is also known as type II interferon.	
Cytokine Production	chemokine biosynthetic process	GO:0042033	The chemical reactions and pathways resulting in the formation of chemokines, any member of a family of small chemotactic cytokines; their name is derived from their ability to induce directed chemotaxis in nearby responsive cells. All chemokines possess a number of conserved cysteine residues involved in intramolecular disulfide bond formation. Some chemokines are considered pro-inflammatory and can be induced during an immune response to recruit cells of the immune system to a site of infection, while others are considered homeostatic and are involved in controlling the migration of cells during normal processes of tissue maintenance or development. Chemokines are found in all vertebrates, some viruses and some bacteria.	
	interleukin-2 biosynthetic process	GO:0042094	The chemical reactions and pathways resulting in the formation of interleukin-2.	
	negative regulation of interferon-gamma production	GO:0032689	Any process that stops, prevents, or reduces the frequency, rate, or extent of interferon-gamma production. Interferon-gamma is also known as type II interferon.	
	positive regulation of chemokine production	GO:0032722	Any process that activates or increases the frequency, rate, or extent of chemokine production.	
	positive regulation of cytokine biosynthetic process	GO:0042108	Any process that activates or increases the frequency, rate or extent of the chemical reactions and pathways resulting in the formation of cytokines.	
	positive regulation of cytokine production involved in immune response	GO:0002720	Any process that activates or increases the frequency, rate, or extent of cytokine production that contributes to an immune response.	
	positive regulation of cytokine secretion	GO:0050715	Any process that activates or increases the frequency, rate or extent of the regulated release of cytokines from a cell.	
	positive regulation of interferon-gamma production	GO:0032729	Any process that activates or increases the frequency, rate, or extent of interferon-gamma production. Interferon-gamma is also known as type II interferon.	
	positive regulation of interleukin-10 production	GO:0032733	Any process that activates or increases the frequency, rate, or extent of interleukin-10 production.	
	positive regulation of interleukin-2 production	GO:0032743	Any process that activates or increases the frequency, rate, or extent of interleukin-2 production.	
	positive regulation of interleukin-6 production	GO:0032755	Any process that activates or increases the frequency, rate, or extent of interleukin-6 production.	
	positive regulation of interleukin-8 production	GO:0032757	Any process that activates or increases the frequency, rate, or extent of interleukin-8 production.	

	positive regulation of tumor necrosis factor production	GO:0032760	Any process that activates or increases the frequency, rate, or extent of tumor necrosis factor production.	
	regulation of interleukin-1 production	GO:0032652	Any process that modulates the frequency, rate, or extent of interleukin-1 production.	
	regulation of interleukin-12 production	GO:0032655	Any process that modulates the frequency, rate, or extent of interleukin-12 production.	

Supplementary Table 6. Complete results of Cox regression analysis in the index cohort for all 64 analyzed GO immune-related processes, sorted by p-value. GO gene ontology; HR hazard ratio; CI confidence interval; *p≤0.05

GO Immune-Related Biological Process	*HR (95% CI)*	*p-value*
response to interferon-gamma	0.53 (0.38 , 0.73)	***≤ 0.001 ****
T cell migration	2.07 (1.43 , 3)	***≤ 0.001 ****
regulation of mononuclear cell migration	0.6 (0.44 , 0.82)	***0.001 ****
monocyte chemotaxis	1.7 (1.22 , 2.38)	***0.002 ****
negative regulation of adaptive immune response	1.73 (1.21 , 2.46)	***0.003 ****
lymphocyte homeostasis	0.59 (0.42 , 0.84)	***0.003 ****
hemopoiesis	0.62 (0.44 , 0.87)	***0.005 ****
production of molecular mediator involved in inflammatory response	1.43 (1.11 , 1.86)	***0.006 ****
negative regulation of inflammatory response	1.43 (1.1 , 1.85)	***0.007 ****
positive regulation of cytokine secretion	0.65 (0.46 , 0.91)	***0.011 ****
B cell activation	1.6 (1.08 , 2.37)	***0.02 ****
positive regulation of leukocyte differentiation	1.77 (1.07 , 2.93)	***0.027 ****
negative regulation of antigen receptor-mediated signaling pathway	0.74 (0.57 , 0.97)	***0.032 ****
T cell costimulation	1.37 (1.02 , 1.85)	***0.039 ****
positive regulation of interleukin-10 production	1.41 (1.01 , 1.96)	***0.041 ****
positive regulation of leukocyte chemotaxis	0.75 (0.57 , 0.99)	***0.041 ****
positive regulation of inflammatory response	0.78 (0.61 , 0.99)	***0.042 ****
positive regulation of leukocyte mediated immunity	1.63 (1.02 , 2.62)	***0.042 ****
regulation of interleukin-1 production	0.65 (0.42 , 0.99)	***0.044 ****
positive regulation of interleukin-6 production	1.36 (1 , 1.85)	*0.052*
positive regulation of lymphocyte activation	0.49 (0.23 , 1.03)	*0.061*
regulation of mast cell activation involved in immune response	0.59 (0.34 , 1.04)	*0.068*
adaptive immune response based on somatic recombination of immune receptors built from immunoglobulin superfamily domains	0.73 (0.53 , 1.02)	*0.068*
immunoglobulin secretion	1.24 (0.98 , 1.58)	*0.078*
positive regulation of tumor necrosis factor production	1.53 (0.9 , 2.59)	*0.112*
negative regulation of leukocyte degranulation	1.4 (0.92 , 2.12)	*0.114*
positive regulation of chemokine production	0.64 (0.37 , 1.12)	*0.118*
interleukin-2 biosynthetic process	0.79 (0.56 , 1.1)	*0.16*
positive regulation of immunoglobulin production	0.71 (0.44 , 1.15)	*0.17*
lymphocyte proliferation	1.39 (0.87 , 2.24)	*0.173*
myeloid cell activation involved in immune response	4.45 (0.49 , 40.26)	*0.184*
positive regulation of cytokine biosynthetic process	1.23 (0.88 , 1.73)	*0.216*
inflammatory response to wounding	1.22 (0.88 , 1.69)	*0.222*
regulation of interleukin-12 production	1.28 (0.86 , 1.9)	*0.225*
chemokine biosynthetic process	1.28 (0.85 , 1.93)	*0.228*
positive regulation of interleukin-8 production	1.21 (0.89 , 1.64)	*0.229*
lymphocyte activation involved in immune response	1.35 (0.83 , 2.2)	*0.231*
activation of innate immune response	0.5 (0.15 , 1.62)	*0.247*
granulocyte chemotaxis	1.21 (0.87 , 1.68)	*0.263*
neutrophil degranulation	0.32 (0.04 , 2.66)	*0.289*
lymphocyte chemotaxis	0.8 (0.52 , 1.23)	*0.309*
regulation of natural killer cell mediated immunity	1.14 (0.86 , 1.52)	*0.369*
microglial cell activation	0.85 (0.6 , 1.21)	*0.371*
innate immune response-activating signal transduction	1.58 (0.56 , 4.51)	*0.388*
positive regulation of cytokine production involved in immune response	0.83 (0.54 , 1.27)	*0.391*
immune response-regulating signaling pathway	1.2 (0.76 , 1.9)	*0.434*
myeloid leukocyte mediated immunity	0.49 (0.08 , 3.05)	*0.448*
negative regulation of lymphocyte activation	0.86 (0.56 , 1.31)	*0.475*
positive regulation of cellular extravasation	0.89 (0.63 , 1.24)	*0.482*
negative regulation of interferon-gamma production	0.91 (0.67 , 1.23)	*0.545*
positive regulation of interferon-gamma production	0.9 (0.61 , 1.31)	*0.567*
regulation of myeloid cell differentiation	1.07 (0.84 , 1.38)	*0.574*
regulation of macrophage activation	1.12 (0.7 , 1.79)	*0.646*
positive regulation of adaptive immune response	0.91 (0.59 , 1.39)	*0.657*
positive regulation of innate immune response	1.18 (0.53 , 2.62)	*0.692*
T cell activation	0.9 (0.53 , 1.53)	*0.692*
neutrophil homeostasis	1.05 (0.77 , 1.44)	*0.758*
lymphocyte differentiation	1.1 (0.58 , 2.09)	*0.77*
natural killer cell mediated immunity	0.97 (0.76 , 1.22)	*0.773*
positive regulation of interleukin-2 production	1.03 (0.75 , 1.43)	*0.839*
granulocyte activation	1.26 (0.13 , 11.74)	*0.84*
negative regulation of leukocyte differentiation	1.02 (0.77 , 1.36)	*0.877*

Supplementary Table 6. Complete results of Cox regression analysis in the index cohort for all 64 analyzed GO immune-related processes, sorted by p-value. GO gene ontology; HR hazard ratio; CI confidence interval; *p≤0.05

GO Immune-Related Biological Process	***HR (95% CI)***	***p-value***
regulation of neuroinflammatory response	1.02 (0.8 , 1.3)	*0.884*

Supplementary Table 7. Complete results of Cox regression analysis in the validation cohort for all 64 analyzed GO immune-related processes, sorted by p-value. GO gene ontology; HR hazard ratio; CI confidence interval; *p≤0.05

GO Immune-Related Biological Process	*HR (95% CI)*	*p-value*
positive regulation of cytokine secretion	0.44 (0.32 , 0.62)	***≤ 0.001 ****
production of molecular mediator involved in inflammatory response	1.48 (1.19 , 1.86)	***≤ 0.001 ****
positive regulation of inflammatory response	0.69 (0.55 , 0.86)	***0.001 ****
positive regulation of leukocyte differentiation	2.62 (1.46 , 4.71)	***0.001 ****
negative regulation of adaptive immune response	1.73 (1.24 , 2.41)	***0.001 ****
positive regulation of interferon-gamma production	0.58 (0.4 , 0.86)	***0.007 ****
positive regulation of chemokine production	0.53 (0.33 , 0.84)	***0.007 ****
regulation of interleukin-12 production	1.61 (1.14 , 2.28)	***0.007 ****
positive regulation of immunoglobulin production	0.56 (0.35 , 0.88)	***0.012 ****
positive regulation of cytokine biosynthetic process	1.48 (1.08 , 2.03)	***0.014 ****
microglial cell activation	1.42 (1.07 , 1.88)	***0.015 ****
regulation of myeloid cell differentiation	0.75 (0.59 , 0.95)	***0.019 ****
T cell costimulation	1.34 (1.01 , 1.77)	***0.043 ****
regulation of natural killer cell mediated immunity	1.36 (1.01 , 1.83)	***0.044 ****
adaptive immune response based on somatic recombination of immune receptors built from immunoglobulin superfamily domains	0.7 (0.49 , 0.99)	***0.046 ****
positive regulation of lymphocyte migration	1.39 (1 , 1.93)	*0.051*
regulation of interleukin-1 production	0.7 (0.47 , 1.06)	*0.09*
positive regulation of interleukin-6 production	1.36 (0.95 , 1.94)	*0.092*
monocyte chemotaxis	1.29 (0.96 , 1.75)	*0.096*
lymphocyte activation involved in immune response	1.56 (0.92 , 2.65)	*0.102*
B cell activation	1.37 (0.93 , 2.01)	*0.107*
regulation of mononuclear cell migration	0.79 (0.6 , 1.06)	*0.113*
neutrophil homeostasis	1.25 (0.93 , 1.68)	*0.133*
negative regulation of leukocyte degranulation	1.34 (0.91 , 1.97)	*0.136*
immune response regulating signaling pathway	1.34 (0.9 , 1.99)	*0.145*
myeloid cell activation involved in immune response	3.96 (0.62 , 25.31)	*0.147*
positive regulation of leukocyte chemotaxis	0.82 (0.63 , 1.08)	*0.154*
regulation of macrophage activation	1.31 (0.89 , 1.92)	*0.168*
positive regulation of interleukin-2 production	0.82 (0.61 , 1.09)	*0.171*
lymphocyte differentiation	0.65 (0.32 , 1.31)	*0.227*
positive regulation of leukocyte mediated immunity	1.32 (0.84 , 2.07)	*0.232*
negative regulation of interferon-gamma production	0.84 (0.64 , 1.12)	*0.238*
neutrophil degranulation	0.43 (0.1 , 1.81)	*0.248*
negative regulation of antigen receptor mediated signaling pathway	0.85 (0.65 , 1.12)	*0.252*
negative regulation of leukocyte differentiation	0.85 (0.63 , 1.14)	*0.266*
myeloid leukocyte mediated immunity	0.42 (0.09 , 2)	*0.274*
positive regulation of interleukin-8 production	0.85 (0.62 , 1.14)	*0.278*
regulation of neuroinflammatory response	1.14 (0.89 , 1.46)	*0.285*
positive regulation of adaptive immune response	0.79 (0.5 , 1.26)	*0.33*
regulation of mast cell activation involved in immune response	0.78 (0.46 , 1.31)	*0.346*
innate immune response activating signal transduction	1.46 (0.66 , 3.2)	*0.346*
positive regulation of tumor necrosis factor production	1.22 (0.79 , 1.88)	*0.366*
positive regulation of cytokine production involved in immune response	1.19 (0.78 , 1.8)	*0.418*
lymphocyte chemotaxis	0.87 (0.62 , 1.23)	*0.426*
response to interferon-gamma	0.9 (0.7 , 1.16)	*0.427*
activation of innate immune response	0.74 (0.29 , 1.86)	*0.517*
positive regulation of interleukin-10 production	1.1 (0.83 , 1.45)	*0.518*
granulocyte chemotaxis	0.91 (0.68 , 1.22)	*0.519*
positive regulation of cellular extravasation	0.91 (0.65 , 1.25)	*0.552*
T cell migration	1.1 (0.8 , 1.5)	*0.57*
negative regulation of inflammatory response	0.94 (0.73 , 1.19)	*0.589*
hemopoiesis	0.92 (0.65 , 1.29)	*0.623*
positive regulation of innate immune response	0.84 (0.39 , 1.82)	*0.654*
natural killer cell mediated immunity	0.95 (0.76 , 1.19)	*0.665*
granulocyte activation	1.39 (0.29 , 6.6)	*0.679*
negative regulation of lymphocyte activation	0.93 (0.64 , 1.36)	*0.713*
lymphocyte homeostasis	0.94 (0.67 , 1.32)	*0.718*
interleukin-2 biosynthetic process	0.96 (0.68 , 1.35)	*0.813*
chemokine biosynthetic process	0.97 (0.7 , 1.33)	*0.829*
lymphocyte proliferation	1.05 (0.66 , 1.66)	*0.842*
T cell activation	0.95 (0.55 , 1.63)	*0.851*
positive regulation of lymphocyte activation	0.94 (0.5 , 1.8)	*0.862*

Supplementary Table 7. Complete results of Cox regression analysis in the validation cohort for all 64 analyzed GO immune-related processes, sorted by p-value. GO gene ontology; HR hazard ratio; CI confidence interval; *p≤0.05

GO Immune-Related Biological Process	*HR (95% CI)*	*p-value*
immunoglobulin secretion	1.01 (0.8 , 1.28)	*0.912*

Supplementary Table 8. Univariable Cox regression for all-cause mortality at 2 years. Results are presented for each of the 187 biomarkers associated with immune-related properties. *p≤0.05, **p-value significant after adjusted for multiple comparisons using the Benjamini-Hochberg test at a false discovery rate of 5%. HR hazard ratio; 95% CI 95% confidence interval

hGNC Abbreviation	Uniprot Identifier	HR (95% CI)	p-value
ADAMTS13	Q76LX8	0.23 (0.17, 0.32)	<0.001**
SMAD5	Q99717	0.39 (0.26, 0.59)	<0.001**
KDR	P35968	0.55 (0.43, 0.69)	<0.001**
CLEC4C	Q8WTT0	0.62 (0.54, 0.71)	<0.001**
SIT1	Q9Y3P8	0.73 (0.62, 0.86)	<0.001**
GDF2	Q9UK05	0.79 (0.7, 0.89)	<0.001**
EGFR	P00533	0.8 (0.66, 0.95)	0.013**
KITLG	P21583	0.81 (0.72, 0.91)	0.001**
ERBB2	P04626	0.83 (0.68, 1.02)	0.072
CLEC4A	Q9UMR7	0.84 (0.7, 1.01)	0.062
IL1RL2	Q9HB29	0.85 (0.71, 1)	0.052
LDLR	P01130	0.87 (0.78, 0.96)	0.007**
MARCO	Q9UEW3	0.89 (0.64, 1.24)	0.504
CCL22	O00626	0.9 (0.82, 0.99)	0.033**
TGM2	P21980	0.9 (0.81, 1.01)	0.082
PDGFB	P01127	0.91 (0.86, 0.97)	0.002**
TLR3	O15455	0.93 (0.82, 1.04)	0.203
LEP	P41159	0.94 (0.88, 1)	0.048*
ANGPT1	Q15389	0.94 (0.88, 1.01)	0.092
GZMB	P10144	0.94 (0.84, 1.06)	0.333
NFATC3	Q12968	0.95 (0.85, 1.07)	0.432
ACP5	P13686	0.96 (0.84, 1.09)	0.54
SRC	P12931	0.97 (0.91, 1.04)	0.344
CD40LG	P29965	0.97 (0.91, 1.04)	0.387
GLB1	P16278	0.98 (0.85, 1.12)	0.727
CASP3	P42574	0.98 (0.93, 1.04)	0.553
DDX58	O95786	0.99 (0.89, 1.09)	0.786
PIK3AP1	Q6ZUJ8	0.99 (0.91, 1.09)	0.899
IL5	P05113	1 (0.92, 1.09)	0.955
FOLR3	P41439	1.01 (0.96, 1.06)	0.772
CCL17	Q92583	1.01 (0.94, 1.08)	0.84
GLO1	Q04760	1.01 (0.91, 1.13)	0.817
ARNT	P27540	1.02 (0.94, 1.1)	0.683
IRAK4	Q9NWZ3	1.02 (0.96, 1.08)	0.479
PECAM1	P16284	1.02 (0.93, 1.12)	0.634
CXCL1	P09341	1.02 (0.95, 1.09)	0.528
LYN	P07948	1.02 (0.94, 1.11)	0.562
FADD	Q13158	1.03 (0.96, 1.11)	0.433
STK4	Q13043	1.03 (0.97, 1.1)	0.309
SERPINE1	P05121	1.03 (0.96, 1.12)	0.377
SH2B3	Q9UQQ2	1.04 (0.97, 1.1)	0.261
PRKCQ	Q04759	1.04 (0.89, 1.21)	0.654
CCL24	O00175	1.04 (0.96, 1.13)	0.367
PRDX1	Q06830	1.04 (0.96, 1.14)	0.319
TEK	Q02763	1.05 (0.82, 1.33)	0.714
TRAF2	Q12933	1.05 (0.93, 1.19)	0.443
TRIM21	P19474	1.06 (0.96, 1.16)	0.248
PARP1	P09874	1.06 (0.97, 1.15)	0.183
MMP9	P14780	1.06 (0.97, 1.16)	0.183
SH2D1A	O60880	1.06 (0.95, 1.19)	0.295
HCLS1	P14317	1.07 (1, 1.14)	0.064

ITGB2	P05107	1.07 (0.95, 1.21)	0.282
TANK	Q92844	1.07 (0.96, 1.19)	0.223
F11R	Q9Y624	1.08 (1.01, 1.15)	0.03**
FCRL3	Q96P31	1.08 (0.87, 1.34)	0.486
JUN	P05412	1.08 (0.92, 1.27)	0.327
IKBKG	Q9Y6K9	1.09 (1.01, 1.18)	0.035**
TXLNA	P40222	1.1 (1.03, 1.18)	0.007**
CDKN1A	P38936	1.1 (1.04, 1.17)	0.002**
PRDX3	P30048	1.11 (1, 1.22)	0.05
IRF9	Q00978	1.11 (0.99, 1.24)	0.083
HEXIM1	O94992	1.11 (1.03, 1.19)	0.006**
CHIT1	Q13231	1.11 (1.04, 1.19)	0.001**
IL6R	P08887	1.11 (0.97, 1.28)	0.121
OLR1	P78380	1.12 (1, 1.24)	0.042*
IL1R2	P27930	1.12 (0.98, 1.28)	0.097
FCGR2B	P31994	1.12 (1.03, 1.22)	0.009**
IL17RA	Q96F46	1.13 (0.99, 1.28)	0.064
ITGAV	P06756	1.13 (0.86, 1.48)	0.38
ZBTB16	Q05516	1.13 (1.04, 1.22)	0.003**
CCL11	P51671	1.15 (0.97, 1.35)	0.104
PLAU	P00749	1.15 (1.02, 1.31)	0.029**
TREML2	Q5T2D2	1.15 (1.03, 1.3)	0.016**
BIRC2	Q13490	1.16 (0.98, 1.36)	0.08
MPO	P05164	1.16 (1.02, 1.31)	0.026**
CDH5	P33151	1.17 (1.03, 1.33)	0.012**
PADI2	Q9Y2J8	1.19 (1.05, 1.34)	0.007**
CD28	P10747	1.19 (1.03, 1.38)	0.02**
TRIM5	Q9C035	1.19 (1.06, 1.34)	0.004**
THPO	P40225	1.2 (1.01, 1.43)	0.044*
CCL2	P13500	1.2 (1.08, 1.34)	0.001**
IRAK1	P51617	1.2 (1.09, 1.33)	<0.001**
ANXA1	P04083	1.2 (1.09, 1.33)	<0.001**
CCL16	O15467	1.2 (1.08, 1.34)	<0.001**
IL1RN	P18510	1.21 (1.1, 1.33)	<0.001**
IL18	Q14116	1.21 (1.07, 1.38)	0.003**
NT5E	P21589	1.22 (1.1, 1.36)	<0.001**
LGALS3	P17931	1.22 (1.08, 1.38)	0.001**
IL10	P22301	1.24 (1.14, 1.34)	<0.001**
ABL1	P00519	1.25 (1.16, 1.34)	<0.001**
CEACAM1	P13688	1.25 (0.83, 1.87)	0.282
MUC16	Q8WXI7	1.25 (1.18, 1.32)	<0.001**
MDK	P21741	1.26 (1.17, 1.35)	<0.001**
RARRES2	Q99969	1.26 (1.08, 1.48)	0.004**
ANPEP	P15144	1.27 (1.12, 1.44)	<0.001**
ICOSLG	O75144	1.29 (1.03, 1.61)	0.026**
MMP12	P39900	1.3 (1.18, 1.43)	<0.001**
IL6	P05231	1.32 (1.24, 1.4)	<0.001**
CLEC4G	Q6UXB4	1.32 (1.1, 1.59)	0.003**
HMOX1	P09601	1.32 (1.11, 1.57)	0.002**
HGF	P14210	1.34 (1.24, 1.44)	<0.001**
MMP3	P08254	1.34 (1.24, 1.46)	<0.001**
KLRD1	Q13241	1.36 (1.19, 1.54)	<0.001**
FABP4	P15090	1.36 (1.27, 1.45)	<0.001**
MB	P02144	1.36 (1.26, 1.47)	<0.001**
VEGFD	O43915	1.37 (1.12, 1.66)	0.002**
ICAM2	P13598	1.38 (1.22, 1.55)	<0.001**
TNFSF13B	Q9Y275	1.38 (1.24, 1.54)	<0.001**

HSPB1	P04792	1.38 (1.14, 1.69)	0.001**
CHI3L1	P36222	1.39 (1.29, 1.5)	<0.001**
CD70	P32970	1.39 (1.22, 1.6)	<0.001**
PGLYRP1	O75594	1.41 (1.27, 1.57)	<0.001**
GRN	P28799	1.43 (1.24, 1.65)	<0.001**
RETN	Q9HD89	1.43 (1.29, 1.58)	<0.001**
AXL	P30530	1.44 (1.27, 1.62)	<0.001**
IL17D	Q8TAD2	1.45 (1.25, 1.68)	<0.001**
LAG3	P18627	1.45 (1.27, 1.66)	<0.001**
CEACAM8	P31997	1.46 (1.33, 1.6)	<0.001**
CTSZ	Q9UBR2	1.47 (1.3, 1.66)	<0.001**
CTSD	P07339	1.47 (1.3, 1.66)	<0.001**
SLAMF7	Q9NQ25	1.49 (1.34, 1.65)	<0.001**
XCL1	P47992	1.49 (1.31, 1.69)	<0.001**
ITM2A	O43736	1.51 (1.31, 1.74)	<0.001**
CD160	O95971	1.52 (1.36, 1.71)	<0.001**
NCR1	O76036	1.53 (1.34, 1.74)	<0.001**
CXCL13	O43927	1.53 (1.4, 1.68)	<0.001**
TNFRSF13B	O14836	1.54 (1.35, 1.75)	<0.001**
CCL15	Q16663	1.55 (1.4, 1.71)	<0.001**
CD93	Q9NPY3	1.55 (1.35, 1.79)	<0.001**
CXCL16	Q9H2A7	1.56 (1.37, 1.77)	<0.001**
IL2RA	P01589	1.56 (1.42, 1.72)	<0.001**
CD83	Q01151	1.57 (1.35, 1.82)	<0.001**
TNFRSF14	Q92956	1.58 (1.43, 1.73)	<0.001**
MERTK	Q12866	1.58 (1.34, 1.85)	<0.001**
IL18BP	O95998	1.58 (1.42, 1.76)	<0.001**
PTX3	P26022	1.59 (1.4, 1.8)	<0.001**
CCL3	P10147	1.59 (1.42, 1.77)	<0.001**
MILR1	Q7Z6M3	1.59 (1.39, 1.81)	<0.001**
IL1R1	P14778	1.59 (1.41, 1.79)	<0.001**
CD84	Q9UIB8	1.6 (1.36, 1.89)	<0.001**
LPL	P06858	1.6 (1.35, 1.9)	<0.001**
CLEC4D	Q8WXI8	1.6 (1.45, 1.77)	<0.001**
IL1RL1	Q01638	1.6 (1.49, 1.73)	<0.001**
SIRPA	P78324	1.61 (1.43, 1.81)	<0.001**
CLEC6A	Q6EIG7	1.62 (1.42, 1.85)	<0.001**
TNFRSF1B	P20333	1.63 (1.49, 1.78)	<0.001**
S100A11	P31949	1.63 (1.37, 1.94)	<0.001**
CLEC7A	Q9BXN2	1.63 (1.47, 1.82)	<0.001**
VIM	P08670	1.68 (1.49, 1.89)	<0.001**
TNFRSF4	P43489	1.68 (1.49, 1.89)	<0.001**
FST	P19883	1.68 (1.45, 1.94)	<0.001**
LY9	Q9HBG7	1.69 (1.39, 2.04)	<0.001**
LTBR	P36941	1.7 (1.54, 1.89)	<0.001**
IL12RB1	P42701	1.79 (1.53, 2.09)	<0.001**
CXCL17	Q6UXB2	1.82 (1.6, 2.08)	<0.001**
LGALS1	P09382	1.82 (1.32, 2.53)	<0.001**
TNFRSF11A	Q9Y6Q6	1.83 (1.66, 2.01)	<0.001**
AGER	Q15109	1.83 (1.55, 2.17)	<0.001**
CKAP4	Q07065	1.86 (1.7, 2.04)	<0.001**
CXCL12	P48061	1.88 (1.33, 2.64)	<0.001**
CXADR	P78310	1.9 (1.71, 2.11)	<0.001**
IGFBP2	P18065	1.93 (1.73, 2.15)	<0.001**
ADAM8	P78325	1.94 (1.62, 2.32)	<0.001**
PLAUR	Q03405	1.96 (1.76, 2.18)	<0.001**
PGF	P49763	2.01 (1.75, 2.3)	<0.001**

IL27	Q8NEV9	2.1 (1.77, 2.51)	<0.001**
LILRB4	Q8NHJ6	2.11 (1.9, 2.36)	<0.001**
BTN3A2	P78410	2.17 (1.87, 2.52)	<0.001**
PDCD1LG2	Q9BQ51	2.18 (1.83, 2.59)	<0.001**
IL4R	P24394	2.2 (1.96, 2.46)	<0.001**
VEGFA	P15692	2.3 (1.99, 2.64)	<0.001**
CTSL	P07711	2.36 (1.99, 2.79)	<0.001**
CD27	P26842	2.39 (2.04, 2.8)	<0.001**
CCN4	O95388	2.39 (2.12, 2.7)	<0.001**
TGFBR2	P37173	2.47 (2.11, 2.89)	<0.001**
F2R	P25116	2.53 (2.12, 3.01)	<0.001**
CD4	P01730	2.57 (2.16, 3.07)	<0.001**
EPHA2	P29317	2.85 (2.47, 3.29)	<0.001**
DLL1	O00548	2.94 (2.38, 3.62)	<0.001**
LGALS9	O00182	3.19 (2.54, 4.01)	<0.001**
GPNMB	Q14956	3.2 (2.25, 4.56)	<0.001**
IFNGR1	P15260	3.46 (2.84, 4.22)	<0.001**
TNFSF13	O75888	3.65 (3, 4.44)	<0.001**
PIGR	P01833	3.87 (1.94, 7.75)	<0.001**
OSCAR	Q8IYS5	4.15 (2.99, 5.77)	<0.001**
SPON2	Q9BUD6	12.2 (7.48, 19.89)	<0.001**
